# Genome-wide comparative analysis of the JmjC gene family in *Setaria italica* and *Setaria viridis* reveals transcriptional divergence and stress-responsive candidates

**DOI:** 10.3389/fpls.2026.1793570

**Published:** 2026-03-27

**Authors:** Wenqing Shi, Mei Ma, Yao Cao, Zhirui Yan, Xinyi Qiao, Yanli Cheng

**Affiliations:** 1 School of Life Science, Shanxi Normal University, Taiyuan, China; 2 School of Life Sciences, Qilu Normal University, Jinan, China

**Keywords:** abiotic stresses, foxtail millet, gene family, green foxtail, Jumonji C

## Abstract

**Introduction:**

Histone demethylation mediated by Jumonji C (JmjC) domain-containing proteins is a pivotal epigenetic mechanism governing plant development and stress adaptation. However, a comprehensive characterization of this family in foxtail millet (*Setaria italica*) and its wild progenitor, green foxtail (*Setaria viridis*), remains lacking.

**Methods:**

In this study, we performed a genome-wide identification of 24 *SiJMJ* and 23 *SvJMJ* genes, classifying them into five conserved subfamilies. Comparative genomics, promoter cis-element analysis, transcription factor binding site prediction, and expression profiling under various abiotic stresses were conducted to characterize their evolutionary and functional divergence.

**Results:**

Comparative genomics revealed extensive collinearity and structural conservation between the two species, yet significant divergence in promoter cis-elements and transcription factor binding sites suggested potential regulatory remodeling during domestication. Expression profiling indicated distinct tissue-specific patterns, particularly in apical meristems and germination. Under abiotic stresses, *SiJMJ17* was significantly upregulated under saline-alkali and cold stresses, whereas *SiJMJ1* exhibited specific upregulation under waterlogging and herbicide treatments.

**Discussion:**

These findings elucidate the evolutionary history of the Setaria JMJ family and highlight candidate demethylases for enhancing stress resilience in C4 cereal crops through epigenetic engineering.

## Introduction

In the face of escalating global climate change, plants are increasingly subjected to a myriad of abiotic stresses, including drought, salinity, extreme temperatures, and heavy metal toxicity, which severely constrain their growth, development, and agricultural productivity (Zhu, 2016). To survive under such adverse conditions, plants have evolved sophisticated molecular networks to perceive stress signals and mount appropriate adaptive responses. Beyond the well-established transcriptional reprogramming mediated by transcription factors, epigenetic regulation has emerged as a crucial and dynamic layer controlling gene expression patterns in response to environmental cues (Kim et al., 2015). Among various epigenetic mechanisms, histone modifications-such as methylation, acetylation, and phosphorylation—play pivotal roles in modulating chromatin structure and accessibility, thereby influencing the transcriptional status of stress-responsive genes (Le et al., 2025).

Histone lysine methylation is a reversible mark associated with both transcriptional activation and repression, depending on the specific residue and methylation state (e.g., mono-, di-, or tri-methylation). Its dynamic balance is tightly regulated by histone methyltransferases (HMTs) and histone demethylases (HDMs) (Khator et al., 2024). The Jumonji C (JmjC) domain-containing proteins represent the largest family of HDMs in plants. These enzymes facilitate the removal of methyl groups from histone lysine residues via an Fe(II)- and α-ketoglutarate-dependent oxidative reaction (Klose et al., 2006). Based on their domain architecture, plant JMJ proteins are classified into several subfamilies, including KDM5/JARID, KDM4/JMJD2, and JMJD6, each exhibiting distinct domain architectures and functions (Zhang et al., 2018).

Accumulating evidence in model and crop plants has highlighted the critical functions of JMJ proteins in developmental processes and abiotic stress tolerance. For instance, in *Arabidopsis thaliana*, JMJ30/JMJD5 is involved in circadian function (Lu et al., 2011; Jones et al., 2019). Similarly, in rice (*Oryza sativa*), OsJMJ704 is a positive regulator of rice bacterial blight resistance (Hou et al., 2015), while OsJMJ713 plays an essential role in heat stress responses in rice (Chai et al., 2024). In maize (*Zea mays*), ZmJMJ703 orchestrates salt stress adaptation (Wang et al., 2025). These studies underscore the conserved yet diversified roles of JMJ-mediated histone demethylation in fine-tuning stress-responsive gene networks across different plant species.

Foxtail millet (*Setaria italica*), a diploid C4 model cereal crop, possesses remarkable inherent tolerance to drought and nutrient-poor soils, making it an excellent system for dissecting the genetic and epigenetic basis of stress resilience (Muthamilarasan and Prasad, 2021). Its small, fully sequenced genome and close genetic relationship to bioenergy grasses like switchgrass further enhance its value as a model system (He et al., 2024). Green foxtail (*Setaria viridis*), the wild progenitor of foxtail millet, exhibits extensive genetic diversity and adaptability to harsh environments (Brutnell et al., 2010; Jia et al., 2013). Comparative genomic studies between these two species provide a unique opportunity to explore the evolutionary divergence and potential domestication signatures of stress-responsive pathways.

To date, genome-wide identification and characterization of the JMJ gene family have been conducted in several key plant species, including *Arabidopsis*, rice, maize, wheat, and soybean (Lu et al., 2008; Han et al., 2016; Qian et al., 2019; Wang et al., 2022). However, despite the agricultural and evolutionary importance of the *Setaria* genus, a comprehensive genome-wide analysis of the JMJ gene family in *S. italica* and *S. viridis* remains unreported. The lack of systematic identification and characterization of these epigenetic regulators hinders our understanding of how histone demethylation contributes to the superior stress tolerance observed in millets. Furthermore, clarifying the evolutionary relationship of JMJ genes between foxtail millet and its wild progenitor is essential to understand the impact of domestication on epigenetic regulatory machinery.

In this study, we performed a comprehensive genome-wide identification and comparative analysis of JmjC domain-containing proteins in both *Setaria italica* and *Setaria viridis*. We systematically analyzed their phylogenetic relationships, gene structures, conserved domains, chromosomal distributions, and promoter *cis*-elements. Moreover, to identify candidate demethylases involved in stress adaptation, we investigated the expression profiles of *SiJMJ* and *SvJMJ* genes under drought, salinity, and cold stresses using transcriptomic data. This study provides a foundational resource for the *Setaria* JMJ gene family and identifies key candidate genes implicated in the epigenetic regulation of abiotic stress tolerance, offering potential targets for improving stress resilience in cereals through epigenetic engineering.

## Materials and methods

### Identification of JmjC genes in foxtail millet and green foxtail

The genome sequences and annotated protein files for foxtail millet (*Setaria italica*, v2.2) and green foxtail (*Setaria viridis*, v2.1) were downloaded from the Phytozome database (Goodstein et al., 2012). To comprehensively identify all putative JmjC domain-containing proteins, a dual strategy was employed. First, the Hidden Markov Model (HMM) profile of the JmjC domain (PF02373) was obtained from the Pfam database (Mistry et al., 2021) and used to scan the proteomes of both species using HMMER 3.3.2 (Eddy, 2011) with a default E-value threshold. Second, the known full-length JMJ protein sequences from *Arabidopsis thaliana* and *Oryza sativa* (Lu et al., 2008) were used as queries for BLASTP searches (E-value < 1e^-5^) against the two Setaria proteomes. All candidate sequences retrieved from both methods were merged. The presence of the conserved JmjC domain was further verified using the SMART (Letunic et al., 2021) InterPro (Blum et al., 2025), and NCBI’s Conserved Domain Database (CDD) (Lu et al., 2020). Redundant sequences and those lacking the complete core domain were manually removed. The final, non-redundant sets were designated as Si.JMJs (*S.italica*) and Sv.JMJs (*S.viridis*).

### Multiple sequence alignment and phylogenetic analysis

Full-length protein sequences of the identified SiJMJs and SvJMJs, along with validated JMJ proteins from Arabidopsis and rice, were aligned using MAFFT (https://toolkit.tuebingen.mpg.de/tools/mafft) with default parameters. A phylogenetic tree was constructed using the Maximum Likelihood method in MEGA 12.0 (Kumar et al., 2024). Branch support was assessed with 1000 bootstrap replicates. The tree was further refined and visualized on the Evolview v3 platform (https://www.evolgenius.info/evolview/) (Subramanian et al., 2019).

### Chromosome location and gene syntenic analysis of JmjC genes in foxtail millet and green foxtail

Chromosomal positions of the JMJ genes were extracted from the GFF3 annotation files. Their physical distribution was mapped using the online tool MapGene2Chromosome v2.1 (http://mg2c.iask.in/mg2c_v2.1/) (Chao et al., 2021). The resulting map was generated with default parameters to visualize the distribution of genes across all chromosomes. Synteny analysis was performed using the MCScanX algorithm integrated with TBtools-II software (version V2.400) (Chen et al., 2023) with default parameters. BLASTP results (E-value < 1e^-10)^ and genome annotation files for both species were used as input to identify syntenic blocks. The syntenic relationships of JMJ gene pairs between and within the two genomes were visualized using the Advanced Circos feature in TBtools-II (Chen et al., 2023). Gene duplication modes (segmental, tandem, etc.) were classified by MCScanX. For each syntenic/duplicated JMJ gene pair, the non-synonymous (Ka) and synonymous (Ks) substitution rates were calculated using TBtools software. On the basis of the resulting Ka/Ks ratios, the gene pairs were categorized as evolving under positive selection (Ka/Ks > 1), neutral evolution (Ka/Ks = 1), or purifying selection (Ka/Ks < 1).

### Prediction of *cis*-acting elements in the promoter region of JmjC genes

For each SiJMJ and SvJMJ gene, the genomic sequence 2000 bp upstream of the transcription start site was extracted. These promoter sequences were submitted to the PlantCARE database (Lescot et al., 2002) for identification of *cis*-regulatory elements. The predicted elements were categorized by function (e.g., stress-responsive, hormone-responsive, light-responsive).

### Transcription factor regulatory network analysis of JmjC genes

To predict potential transcriptional regulators of the Setaria JMJ genes, the promoter sequences (2000 bp upstream) were analyzed using Plant Transcriptional Regulatory Map (PTRM, http://plantregmap.gao-lab.org/) (Tian et al., 2020), with *Arabidopsis thaliana* as the reference species and a screening threshold of P ≤ 1e^-5^.

### Prediction of JmjC protein structures

The secondary structures of SiJMJ and SvJMJ proteins were predicted using the FELLS (Fast Estimator of Latent Local Structure) server (http://old.protein.bio.unipd.it/fells/) with default parameters. For tertiary structure prediction, homology modeling was performed using the SWISS-MODEL server (https://swissmodel.expasy.org/). Template search was carried out against the SWISS-MODEL template library using BLAST and HHblits. For each target, multiple models were generated based on different templates, and the model with the highest Global Model Quality Estimation (GMQE) score was selected as the final tertiary structure. GMQE is a combined score that estimates the expected accuracy of the model based on template coverage and target-template sequence identity. The quality of the selected models was further evaluated by the QMEAN (Qualitative Model Energy Analysis) scoring function, which provides a global and local estimate of model reliability.

### Analysis of the expression patterns of JmjC genes in different tissues

The expression data of the Setaria JMJ genes in different tissues at different stages were obtained from *Setaria*-db (http://111.203.21.71:8000/index.html). A heatmap of the FPKM values was generated using the pheatmap package in R to visualize tissue-specific expression profiles.

### Expression analysis of JMJ genes under different abiotic stresses

To investigate the expression patterns of *SiJMJ* and *SvJMJ* genes under various abiotic stresses, we retrieved raw RNA-seq datasets from the NCBI Sequence Read Archive (SRA) database. The experimental conditions for the public datasets used in this study are briefly described as follows, based on the metadata associated with each BioProject:

#### Saline-alkali stress (BioProject: PRJNA1168149)

As described in the source dataset, *S. italica* seedlings at the three-leaf stage were treated with 75% seawater (approx. 2.7% salinity) or distilled water (control). Leaf samples were collected at 0, 12, and 24 hours post-treatment (Yang et al., 2025).

#### Drought stress (BioProject: PRJNA1155684)

Seedlings were grown in soil mix (3:1 soil:vermiculite) under standard conditions (28 °C/20 °C). Drought stress was imposed by withholding water at the five-leaf stage until visible wilting occurred, compared to well-watered controls (Chang et al., 2024).

#### Cold stress (BioProject: PRJNA767196)

Seedlings were subjected to 4 °C low-temperature treatment. Samples were collected from plants treated for 2 h, 24 h, and 168 h, alongside a 25 °C control group, as previously described by Nie et al (Nie et al., 2022).

#### Herbicide stress (BioProject: PRJNA751769)

*S. italica* seedlings were grown in a greenhouse and treated at the three-leaf stage with atrazine (2 mL/L of commercial formulation) or water. Leaves were harvested post-treatment to analyze the response to chemical stress (Sun et al., 2022).

#### Heat stress (BioProject: PRJNA896540)

*S. viridis* (genotype A10) seedlings were grown under a 28 °C/20 °C cycle. Two-week-old plants were subjected to heat stress at 42 °C (day)/32 °C (night), while control plants were maintained at standard temperatures. Leaf tissues were collected for sequencing (Zhang et al., 2025).

#### Additional stresses

Datasets for low nitrogen (PRJNA1083237) and waterlogging (PRJNA1074412) were also downloaded and analyzed to broaden the scope of stress response profiling.

Data Processing: Raw reads were filtered to remove low-quality sequences and adapters using Trimmomatic. The clean reads were aligned to the reference genomes of *S. italica* (v2.2) and *S. viridis* (v2.1) using HISAT2. Gene expression levels were calculated as Fragments Per Kilobase of transcript per Million mapped reads (FPKM). Differential expression analysis was performed using DESeq2 (Love et al., 2014), with a threshold of |log2(Fold Change)| ≥ 1 and FDR < 0.05.

## Results

### Genome-wide identification and genomic distribution of JmjC genes in foxtail millet and green foxtail

To comprehensively identify the JmjC gene family in *Setaria*, we performed a genome-wide search using both HMM profiles and BLASTP homology searches. After removing redundant sequences and validating the presence of the core JmjC domain, a total of 24 and 23 JmjC genes were identified in the genomes of foxtail millet (*S.italica*) and green foxtail (*S.viridis*), respectively. These genes were renamed as *SiJMJ1* to *SiJMJ24* and *SvJMJ1* to *SvJMJ23* based on their physical order on the chromosomes (**Supplementary Table 1**). No homologous gene corresponding to *SiJMJ16* was found in *S.viridis*.

**Figure 1:**
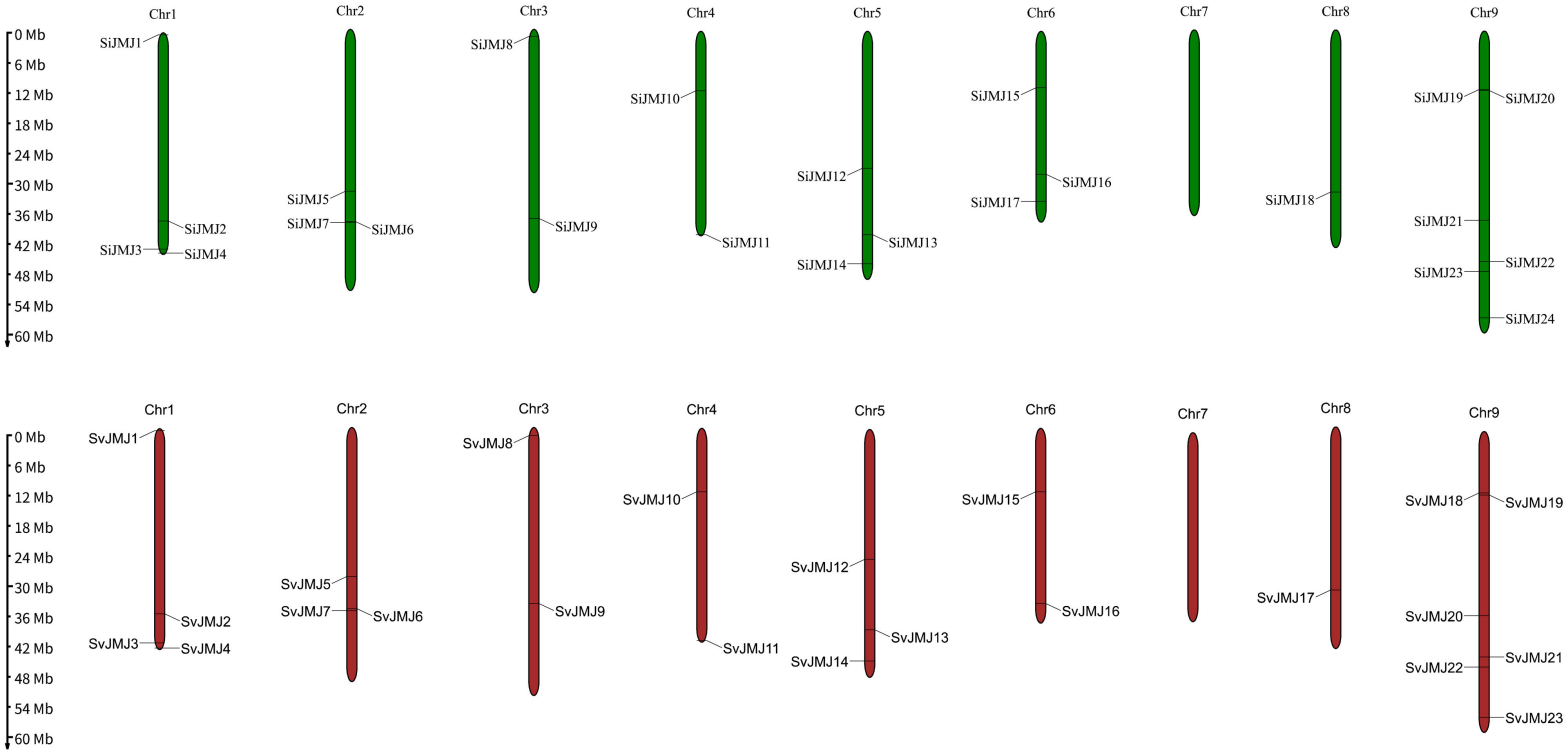
Chromosomal distribution of *JmjC* genes in *Setaria italica* and *Setaria viridis*. The genomic locations of *SiJMJ* genes (top panel) and *SvJMJ* genes (bottom panel) were mapped onto the nine chromosomes of foxtail millet and green foxtail, respectively. The chromosome numbers (Chr1-Chr9) are indicated at the top of each bar. The scale on the left represents the physical length of chromosomes in megabases (Mb). Green bars represent the chromosomes of *S. italica*, and red bars represent the chromosomes of *S.viridis*.

The chromosomal distribution analysis revealed that the JmjC genes were unevenly distributed across the 9 chromosomes of both species (**Figure 1**). In foxtail millet, Chromosome 9 harbored the largest number of JmjC genes (five genes), followed by Chromosome 1 (four genes), whereas Chromosome 8 contained only one and Chromosome 7 had no JmjC genes. A similar distribution pattern was observed in green foxtail, indicating a conserved chromosomal organization between the crop and its wild progenitor. Although most genes were scattered individually, we observed three gene clusters (Chromosome 1, Chromosome 2, and Chromosome 9) where two genes were located within a short physical distance, potentially indicating local duplication events.

### Phylogenetic relationships and evolutionary history of *Setaria* JMJ proteins

**Figure 2:**
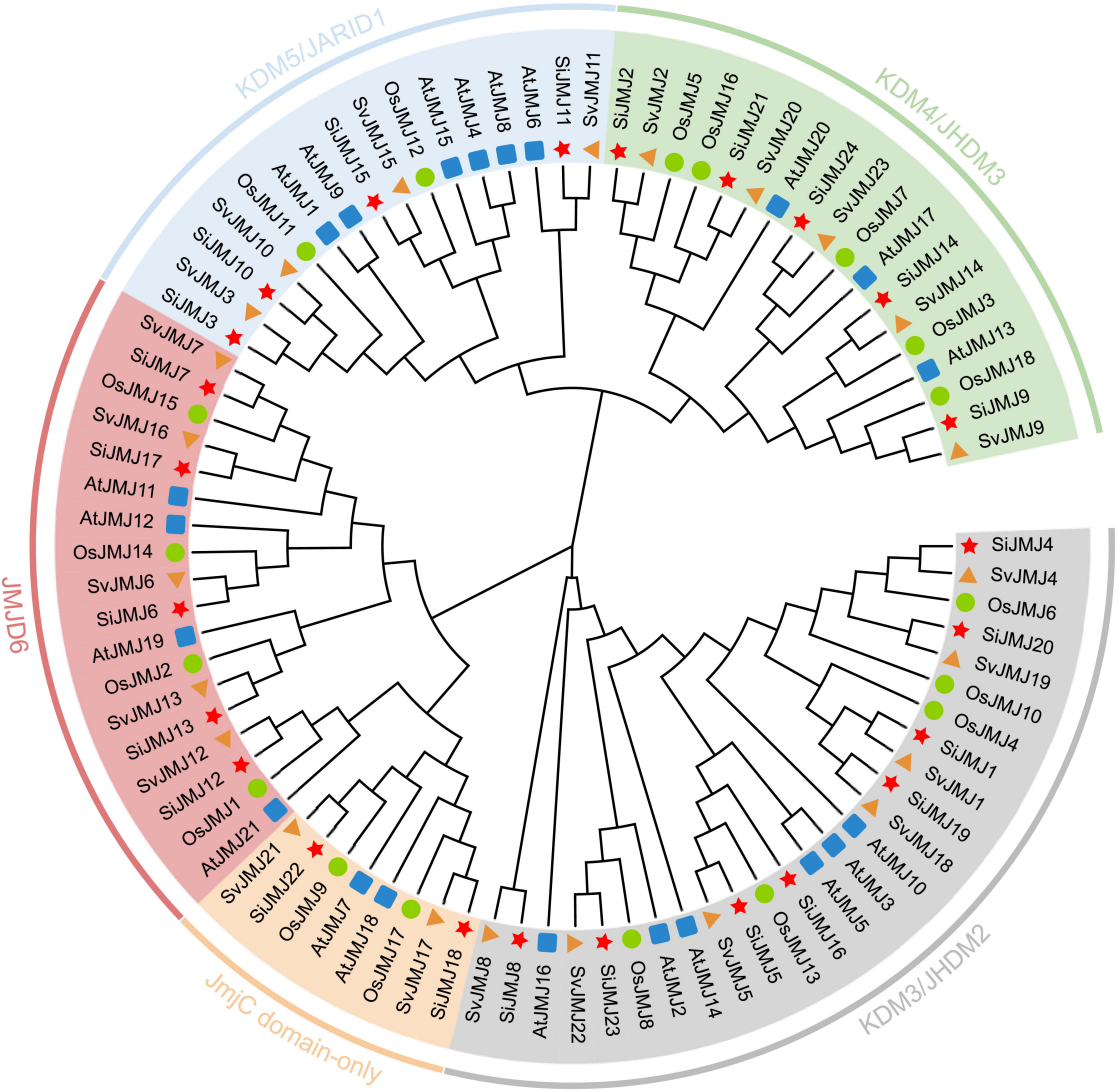
Phylogenetic relationship analysis of JmjC proteins in *Setaria italica*, *Setaria viridis*, *Oryza sativa*, and *Arabidopsis thaliana*. The phylogenetic tree was constructed using the Maximum Likelihood (ML) method with 1000 bootstrap replicates. The JmjC proteins are clustered into five distinct subfamilies: KDM5/JARID1, KDM4/JHDM3, JMJD6, JmjC domain-only, and KDM3/JHDM2, which are distinguished by different background colors. The geometric shapes preceding the gene names indicate the species origin: red stars for *S.italica* (SiJMJs), orange triangles for *S.viridis* (SvJMJs), green circles for *O.sativa* (OsJMJs), and blue squares for *A. thaliana* (AtJMJs).

To elucidate the evolutionary trajectory and functional divergence of the *JmjC* gene family, a phylogenetic tree was constructed using full-length protein sequences from *S.italica*, *S.viridis*, and two representative model species: *Arabidopsis thaliana* (dicot) and *Oryza sativa* (monocot). The phylogenetic topology resolved the JMJ proteins into five distinct and well-conserved subfamilies: KDM5/JARID1, KDM4/JMDM3, KDM3/JMDM2, JMJD6, and JmjC-only (**Figure 2**). The distribution of *Setaria JmjC* genes across these subfamilies was uneven. The KDM3/JMDM2 subfamily was the largest, containing eight *SiJMJ* and seven *SvJMJ* members, whereas the JmjC-only clade was the smallest. This classification pattern is highly consistent with that observed in *Arabidopsis* and rice, suggesting that the diversification of the JMJ family predates the monocot-dicot divergence. In terms of evolutionary relationships, the *Setaria* JMJ proteins (SiJMJs and SvJMJs) consistently clustered more closely with their rice orthologs than with those from *Arabidopsis*. This clustering reflects the established taxonomic relationship within the *Poaceae* family. Notably, a vast majority of *SiJMJ* genes formed one-to-one orthologous pairs with *SvJMJ* genes with high bootstrap support (typically >90%), indicating an extremely high degree of conservation between foxtail millet and its wild progenitor. Furthermore, we identified several lineage-specific duplication events. For instance, in the KDM5/JARID1(*SiJMJ8/SvJMJ8*) and KDM3/JMDM2 (*SiJMJ12/SvJMJ12*) subfamily, *Setaria*-specific sister gene pairs were observed that lacked direct one-to-one orthologs in rice. These findings suggest that while the core JMJ family structure is ancient and conserved, specific subfamilies have undergone recent expansions or functional specializations in the *Setaria* lineage following its divergence from the common ancestor with rice.

### Motif and gene structure analysis of JmjC genes in foxtail millet and green foxtail

**Figure 3:**
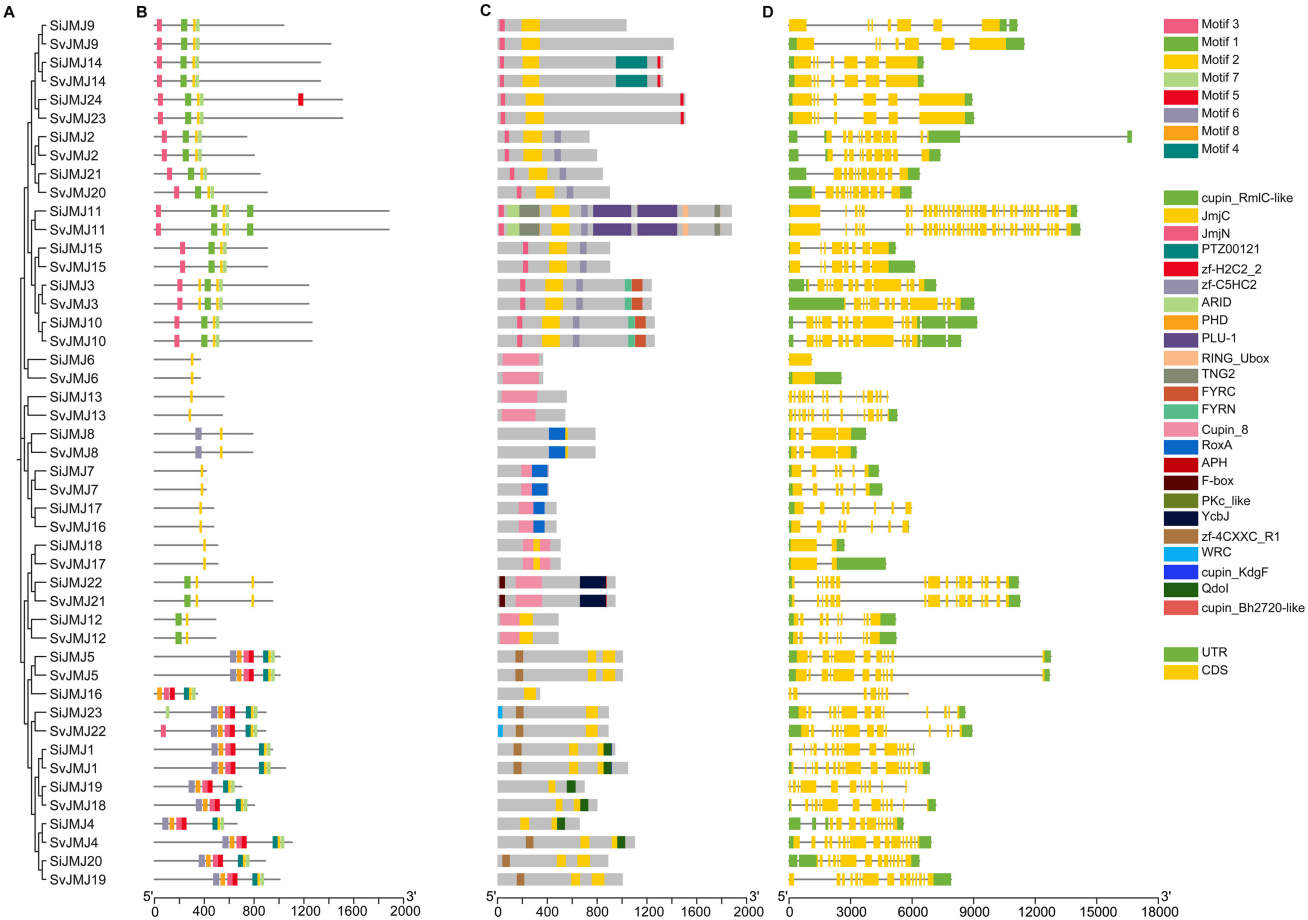
Phylogenetic relationships, conserved motifs, domain architectures, and gene structures of JmjC genes in *Setaria italica* and *Setaria viridis*. **(A)** Phylogenetic tree of SiJMJ and SvJMJ proteins. The tree was constructed to show the evolutionary relationships between foxtail millet and green foxtail orthologs. **(B)** Distribution of conserved motifs in JMJ proteins. The colored boxes represent different conserved motifs (Motifs 1–8) identified by MEME analysis, with specific annotations shown in the right legend. **(C)** Conserved domain compositions. Distinct colored blocks indicate specific functional domains (e.g., JmjC, JmjN, zf-C5HC2, etc.) predicted by the CDD and SMART databases. **(D)** Exon-intron structures of *SiJMJ* and *SvJMJ* genes. Yellow boxes indicate coding sequences (CDS), green boxes indicate untranslated regions (UTRs), and black lines represent introns. The scale bars at the bottom indicate protein length (amino acids) for B and C, and genomic sequence length (base pairs) for D.

To further explore the structural diversity and evolutionary conservation of the JMJ gene family, the conserved protein motifs and exon-intron organizations of all identified *SiJMJ* and *SvJMJ* genes were analyzed based on their phylogenetic relationships (**Figure 3**). The MEME analysis identified a total of eight conserved motifs across the JMJ proteins of both species (**Figure 3B**). As expected, Motif 1, Motif 2 and Motif 7, which correspond to the core JmjC domain (**Figures 3B, C**), were widely present in most members, indicating the functional conservation of histone demethylase activity. Generally, JMJ proteins clustered within the same subfamily shared similar motif compositions and arrangements. For instance, the KDM4/JMDM3 subfamily specifically contained Motif 3 (related to the JmjN domain), whereas the JMJD6 subfamily lacked this motif (**Figures 3B, C**). This subfamily-specific motif distribution suggests that JMJ proteins may have diversified in function during evolution while retaining their core enzymatic mechanisms.

The exon-intron structures of *SiJMJ* and *SvJMJ* genes were examined to gain insights into their structural evolution. A high degree of divergence was observed in the number of exons, which ranged from 1 to 33. However, the gene structure patterns were highly conserved within specific subfamilies. For example, members of the JMJD6 group typically possessed fewer introns (ranging from 1 to 15), whereas the KDM4/JMDM3 group exhibited more complex structures with a higher number of exons. Notably, orthologous gene pairs between foxtail millet and green foxtail (e.g., *SiJMJ14* and *SvJMJ14*) displayed nearly identical exon-intron organizations and motif patterns, further supporting the close evolutionary relationship and functional conservation between the domesticated crop and its wild progenitor. Overall, the consistency between the phylogenetic classification, motif composition, and gene structure provides strong evidence for the reliable identification and classification of JmjC genes in *Setaria*.

### Gene duplication and collinearity analysis of JmjC genes in foxtail millet and green foxtail

**Figure 4:**
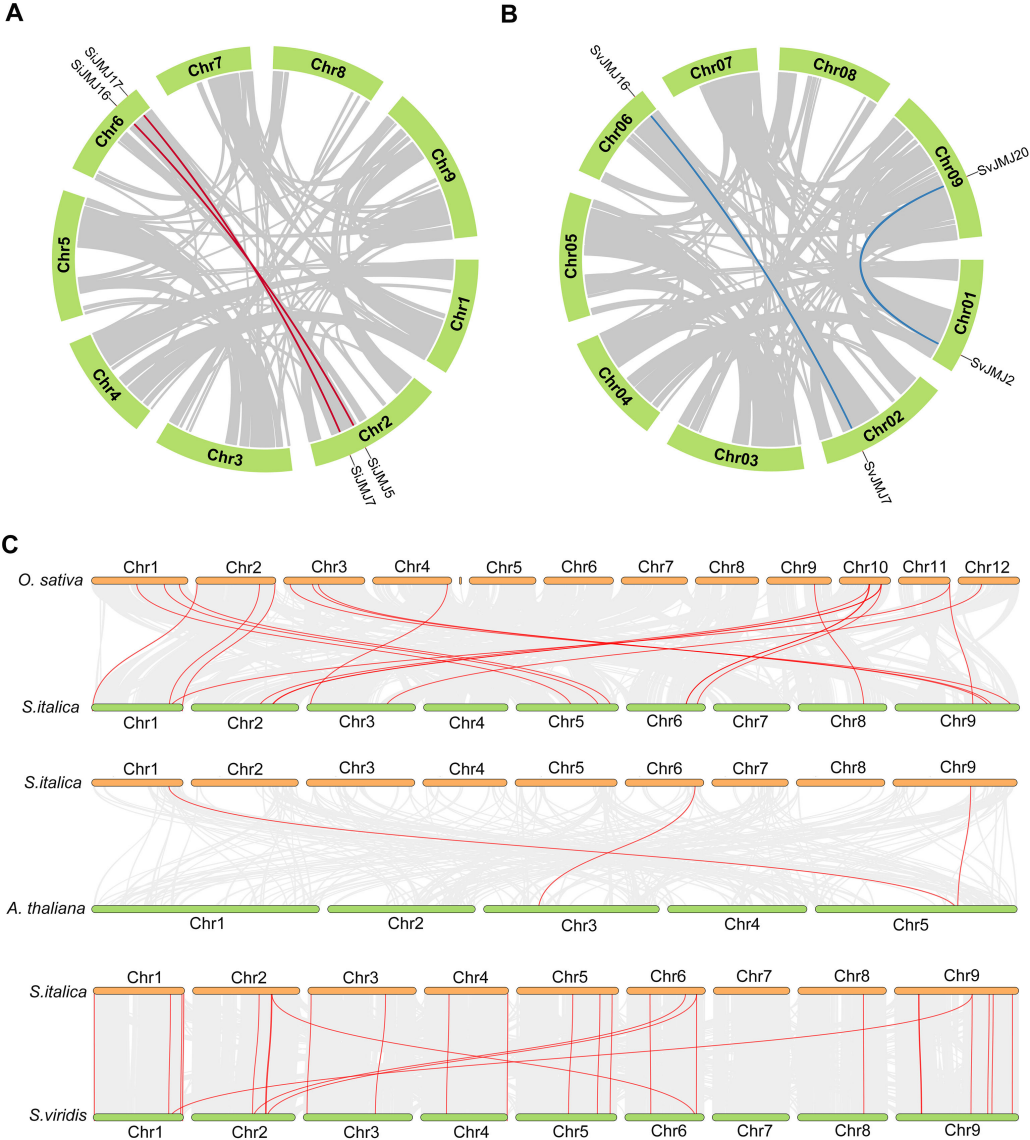
Synteny analysis and chromosomal duplication of *JmjC* genes. **(A, B)** Circos diagrams illustrating segmental duplications of JmjC genes in *Setaria italica***(A)** and *Setaria viridis***(B)**. The chromosomes are represented by green circular blocks. Gray lines in the background indicate collinear blocks within the whole genomes, while red lines in **(A)** and blue lines in **(B)** highlight the duplicated *JmjC* gene pairs. **(C)** Synteny analysis of JmjC genes between *S.italica* and three other plant species (*Oryza sativa*, *Arabidopsis thaliana*, and *S.viridis*). The gray background lines represent the collinear blocks between the indicated genomes, and the red lines highlight the syntenic *JmjC* gene pairs.

Gene duplication is a fundamental driving force in the evolution of gene families and the acquisition of new functions. To elucidate the expansion mechanisms of the *Setaria* JMJ family, we analyzed the duplication events within and between the *S.italica* and *S.viridis* genomes (**Figure 4**). Within the *S. italica* genome, the analysis of duplication modes (using MCScanX) demonstrated that two gene pairs with segmental duplication (or whole-genome duplication, WGD) were identified in the foxtail millet JMJ family (**Figure 4A**). A similar pattern was observed in *S.viridis* (**Figure 4B**).

To explore the evolutionary trajectory of JMJ genes across plant lineages, we analyzed the syntenic relationships between *S.italica* and two representative model species: *Oryza sativa* (rice, monocot) and *Arabidopsis thaliana* (dicot) (**Figure 4C**). Extensive collinearity was observed between *S.italica* and *O.sativa*. A total of 19 orthologous JMJ gene pairs were identified between the two genomes. This high degree of synteny indicates that JMJ genes were well-established in the common ancestor of cereals before the divergence of millet and rice. In contrast, the syntenic relationship with the dicot *A. thaliana* was much weaker, with only three orthologous pairs identified. The significant disparity in syntenic pairs aligns with the phylogenetic classification and reflects the ancient divergence between monocots and dicots. Finally, to assess the genomic conservation between the domesticated crop and its wild progenitor, we performed a comparative collinearity analysis between *S.italica* and *S.viridis*. As expected, the two genomes exhibited an extremely high degree of collinearity. A total of 23 *SiJMJ* genes showed one-to-one orthologous relationships with *SvJMJ* genes, located in highly conserved syntenic blocks (**Figure 4C**). This near-perfect collinearity confirms that the genomic organization of the JMJ family has remained stable and was largely preserved during the domestication of foxtail millet from green foxtail.

To understand the evolutionary driving forces acting on the *Setaria* JMJ gene family, we calculated the non-synonymous (Ka) and synonymous (Ks) substitution rates and their ratios (Ka/Ks) for orthologous gene pairs between *S.viridis* and *S.italica*, as well as for paralogous pairs derived from duplication events (**Supplementary Table 2**). The analysis revealed that the Ka/Ks ratios for the vast majority of gene pairs were significantly less than 1, ranging from 0 to 0.74. This indicates that the JMJ gene family has primarily evolved under strong purifying selection. Notably, several orthologous pairs, such as SvJMJ5 vs. SiJMJ5, SvJMJ6 vs. SiJMJ6, SvJMJ9 vs. SiJMJ9, and SvJMJ13 vs. SiJMJ13, exhibited a Ka value of 0, implying that their amino acid sequences are identical and have been strictly conserved during the divergence of foxtail millet from its wild progenitor. Similarly, all identified paralogous pairs within the genomes (e.g., SvJMJ2 vs. SvJMJ20, SiJMJ5 vs. SiJMJ16) showed low Ka/Ks ratios with a mean of approximately 0.20, suggesting that duplicated genes have been maintained by purifying selection to preserve essential functions.

Despite the overall conservation, two specific orthologous pairs exhibited Ka/Ks ratios greater than 1, providing strong evidence of positive selection: *SvJMJ18*/*SiJMJ19* (Ka/Ks 1.38) and *SvJMJ22*/*SiJMJ23* (Ka/Ks 1.80). These ratios suggest that these specific loci accumulated non-synonymous mutations at a rate faster than neutral expectations. This implies that these genes may have undergone adaptive evolution, potentially contributing to specific agronomic traits or environmental adaptations acquired during the domestication of foxtail millet (*S.italica*) from green foxtail (*S.viridis*).

### Analysis of *cis*-acting elements in the promoter regions of JmjC genes in foxtail millet and green foxtail

**Figure 5:**
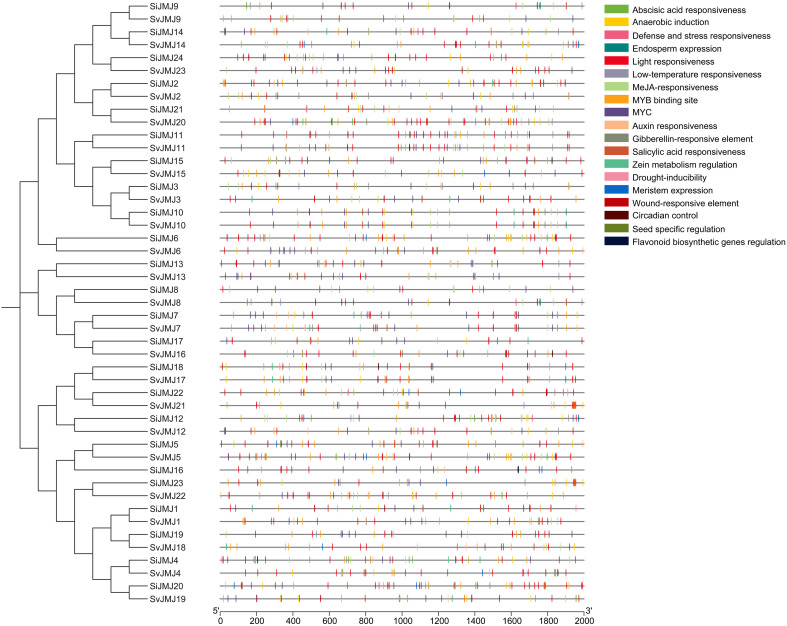
Predicted *cis*-acting elements in the promoter regions of *SiJMJ* and *SvJMJ* genes. The phylogenetic relationship of *SiJMJ* and *SvJMJ* gene pairs is shown on the left. The 2,000 bp upstream genomic sequences relative to the translation start site (TSS) were analyzed for promoter element identification. Different colored vertical bars represent various *cis*-acting elements associated with environmental stress responses (e.g., low-temperature, drought, defense), hormone signaling (e.g., abscisic acid, MeJA, salicylic acid), and plant development (e.g., light responsiveness, meristem expression). The specific functions corresponding to each color are listed in the legend on the right.

**Figure 6:**
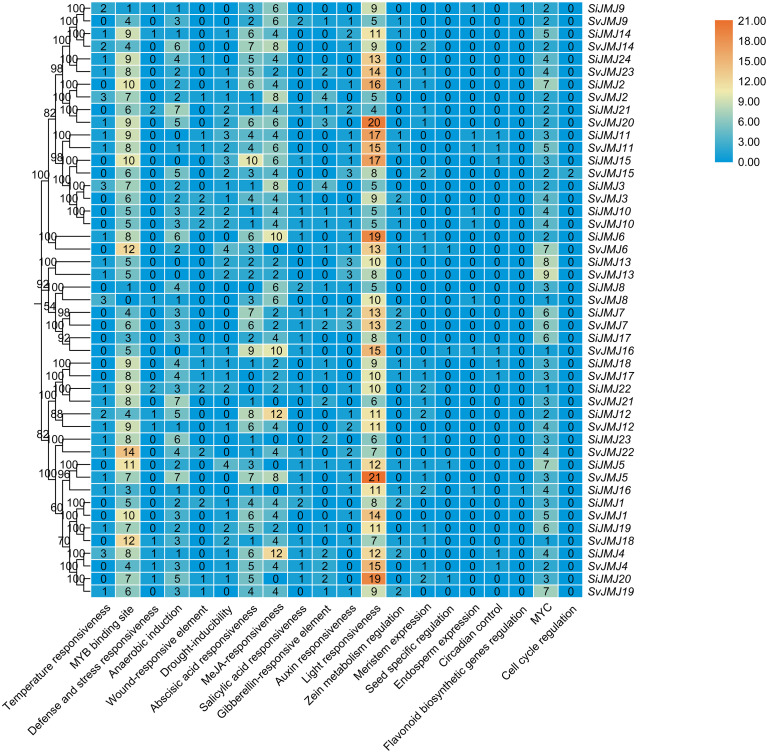
Statistical analysis of *cis*-acting elements in the promoter regions of *SiJMJ* and *SvJMJ* genes. The phylogenetic relationship of *SiJMJ* and *SvJMJ* pairs is shown on the left, with bootstrap values indicated at the nodes. The heatmap displays the abundance of predicted *cis*-acting elements in the 2,000 bp upstream promoter regions. The numbers inside the grid represent the specific count of each element type for each gene. The elements are categorized into environmental stress responsiveness (e.g., temperature, drought, anaerobic), hormone signaling (e.g., abscisic acid, MeJA, auxin), and growth and development (e.g., light responsiveness, meristem expression). The color scale on the right indicates the frequency of elements, ranging from blue (low abundance) to orange (high abundance).

To investigate the potential regulatory mechanisms controlling the expression of *Setaria JMJ* genes, we analyzed the *cis*-acting regulatory elements in the 2000 bp upstream promoter regions of all identified *SiJMJ* and *SvJMJ* genes using the PlantCARE database. A diverse array of *cis*-elements was identified and categorized into three major functional groups: phytohormone responsiveness, abiotic stress responsiveness, and plant growth and development (**Figure 5**; **Supplementary Tables 3**, **5**). Hormone-related elements constituted a significant proportion of the identified motifs. Notably, the Abscisic Acid Responsive Element (ABRE) was the most abundant motif, present in the promoters of 22 *SiJMJ* and 22 *SvJMJ* genes (**Figure 6**; **Supplementary Tables 4**, **6**). Additionally, elements responsive to methyl jasmonate (MeJA) (CGTCA-motif and TGACG-motif), salicylic acid (SA) (TCA-element), and auxin (TGA-element) were widely distributed. In alignment with the strong stress tolerance of *Setaria*, various stress-responsive elements were identified. The MBS (MYB binding site), which is involved in drought inducibility, was found in 24 *SiJMJ* and 22 *SvJMJ* genes (**Figure 6**). The LTR (low-temperature responsiveness) element was identified in 13 *SiJMJ* and 11 *SvJMJ* genes. Other stress-related motifs, such as TC-rich repeats (defense and stress responsiveness) and ARE (anaerobic induction), were also unevenly distributed across the family members (**Figure 6**). For instance, *SiJMJ4* and *SvJMJ2* contained multiple copies of MBS and LTR elements. Apart from stress and hormone regulation, numerous elements related to light responsiveness (e.g., G-box, Box 4, and GT1-motif) were ubiquitous across almost all promoters. Furthermore, tissue-specific motifs such as the CAT-box (related to meristem expression) and O2-site (related to zein metabolism regulation) were present in specific genes. To elucidate the impact of domestication on transcriptional regulation, we further compared the promoter architectures of orthologous gene pairs. While the core developmental elements (e.g., G-box) were largely conserved, divergence was observed in stress-responsive motifs. Notably, the promoters of several *S.viridis* genes (e.g., *SvJMJ6*, *SvJMJ18*, and *SvJMJ22*) exhibited a higher density of MBS elements compared to their *S.italica* orthologs (e.g., *SiJMJ6*, *SiJMJ19*, and *SiJMJ23*) (**Figure 6**). Furthermore, compared to *SvJMJ8*, the promoter of *SiJMJ8* lacked LTR element. This differential accumulation of regulatory motifs implies that the wild progenitor may possess a more dynamic transcriptional response system to adapt to fluctuating natural environments, a trait that might have been partially relaxed during the domestication of foxtail millet.

### Prediction of transcriptional regulators and regulatory network analysis of JmjC genes in foxtail millet and green foxtail

**Figure 7:**
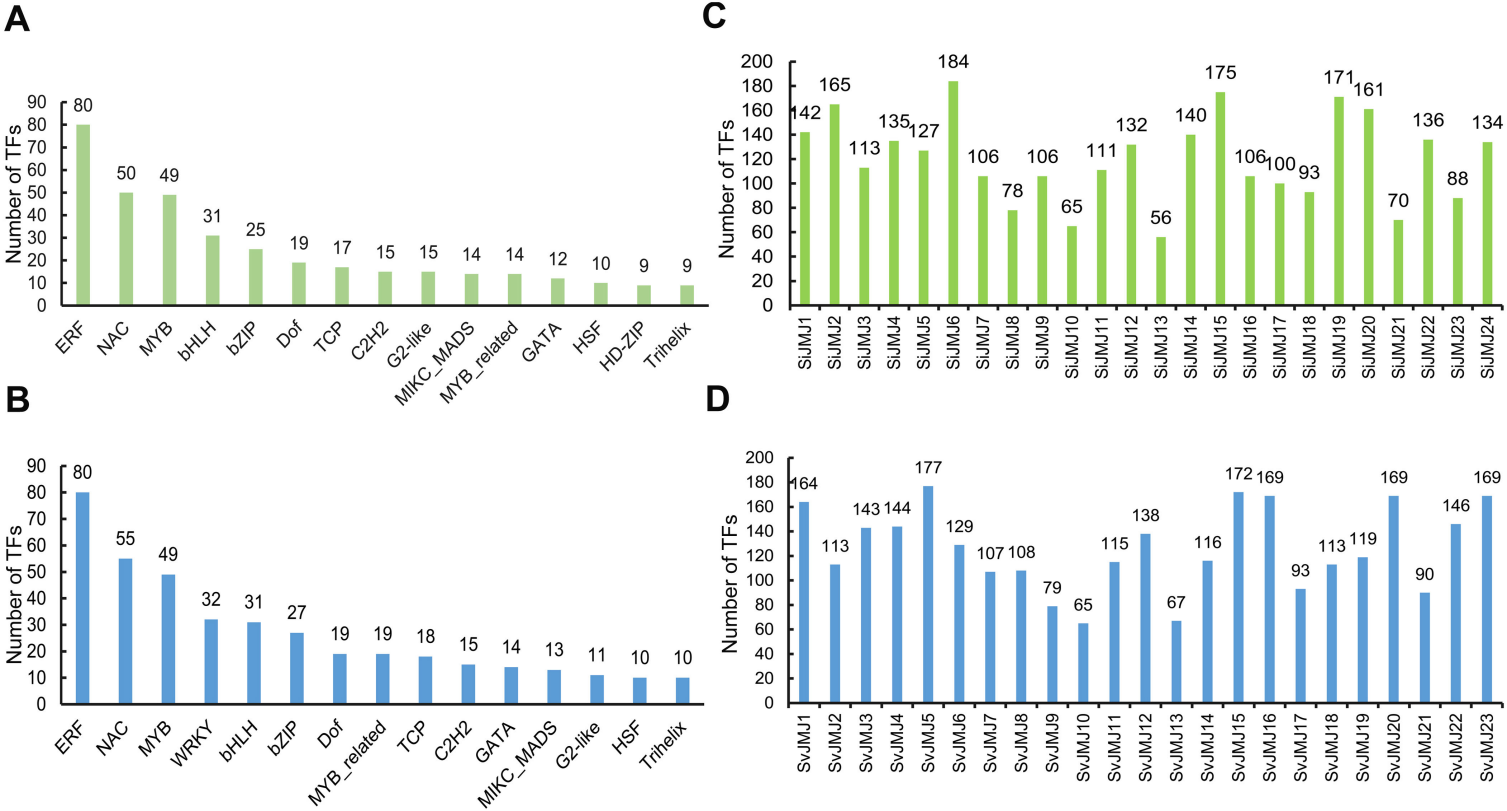
Prediction of transcription factors (TFs) targeting *SiJMJ* and *SvJMJ* promoters. **(A, B)** Frequency distribution of the top transcription factor families predicted to bind to the promoter regions of *SiJMJ* genes **(A)** and *SvJMJ* genes **(B)**. The x-axis represents the TF families (e.g., ERF, NAC, MYB), and the y-axis indicates the number of predicted TFs. **(C, D)** The total count of predicted TFs targeting each individual *SiJMJ* gene **(C)** and *SvJMJ* gene **(D)**. Green bars represent data for *Setaria italica*, and blue bars represent data for *Setaria viridis*. The numbers above the bars indicate the specific count of TFs for each gene.

To elucidate the upstream regulatory mechanisms controlling the *JMJ* gene family, we constructed a transcriptional regulatory network by predicting transcription factors (TFs) targeting the promoter regions of both *SiJMJ* and *SvJMJ* genes using the Plant Transcriptional Regulatory Map (PTRM) database. A total of 455 and 494 putative TFs belonging to 42 families were identified as potential regulators of *SiJMJ* and *SvJMJ* genes, respectively (**Supplementary Tables 7**, **8**). A diverse array of TFs was identified, showing varying abundances across different TF families (**Figure 7**). In foxtail millet (*S.italica*), the identified TFs were classified into multiple families. The ERF (Ethylene Response Factor) family was the most abundant, with 80 members targeting *SiJMJ* genes, followed by the NAC (50) and MYB (49) families (**Figure 7A**). Other significant families included bHLH (31), bZIP (25), and Dof (19). A similar distribution pattern was observed in green foxtail (*S.viridis*). The ERF family remained the dominant regulator (80 members), followed by NAC (55) and MYB (49) (**Figure 7B**). Interestingly, the WRKY family appeared more prominent in the *S.viridis* regulatory network (32 members) compared to *S.italica* (6 members), suggesting subtle divergences in stress-responsive regulation between the crop and its wild progenitor. The predominance of these stress-related TF families (ERF, MYB, NAC, bZIP) implies that *JMJ* genes in *Setaria* are likely heavily regulated by environmental stress signals.

The number of TFs targeting individual *JMJ* genes varied significantly, indicating different levels of transcriptional regulation complexity. In *S.italica*, *SiJMJ6* was identified as the most heavily regulated gene, targeted by 184 TFs, followed by *SiJMJ15* (175) and SiJMJ19 (171) (**Figure 7C**). In contrast, *SiJMJ13* had the fewest regulatory inputs, with only 56 predicted TFs. Similarly, in *S. viridis*, high variability was observed (**Figure 7D**). *SvJMJ5* recruited the highest number of TFs (177), closely followed by SvJMJ15 (172) and a group of genes including *SvJMJ20*, *SvJMJ23*, and *SvJMJ16* (169 each). Conversely, *SvJMJ10* and *SvJMJ13* were targeted by fewer TFs (65 and 67, respectively).

To further explore the evolutionary divergence of transcriptional regulation, we compared the number of predicted TFs between identified orthologous gene pairs of *S.italica* and *S.viridis*. The analysis revealed significant regulatory rewiring during the divergence of the two species. For several gene pairs, the domesticated *S.italica* genes recruited significantly more transcriptional regulators than their wild counterparts, suggesting an expansion of regulatory complexity in the crop lineage. Notably, *SiJMJ6* is targeted by 184 TFs, whereas its ortholog *SvJMJ6* is targeted by only 129. A similar trend was observed for *SiJMJ2* (165 TFs) versus *SvJMJ2* (113 TFs) (**Figures 7C, D**). Furthermore, in the cluster of *SiJMJ19/SvJMJ18* and *SiJMJ20/SvJMJ19*, the *S.italica* genes consistently showed higher TF numbers (171 vs. 113, and 161 vs. 119, respectively), indicating that these loci may have acquired additional regulatory inputs to support specific agronomic traits in foxtail millet.

Conversely, a distinct set of orthologous pairs exhibited a more robust regulatory network in the wild progenitor *S.viridis*, potentially reflecting the need for high plasticity to survive in harsh natural environments. The most striking divergence was observed in the *SiJMJ21/SvJMJ20* pair, where the wild ortholog (*SvJMJ20*) is targeted by 169 TFs, compared to only 70 for *SiJMJ21* in foxtail millet. Similarly, *SvJMJ5* (177 TFs) and *SvJMJ16* (169 TFs) showed a much higher degree of regulation than their respective orthologs *SiJMJ5* (127 TFs) and *SiJMJ17* (100 TFs) (**Figures 7C, D**). Other pairs following this pattern include *SiJMJ3/SvJMJ3* (113 vs.143), *SiJMJ22/SvJMJ21* (136 vs.90), *SiJMJ23/SvJMJ22* (88 vs.146), and *SiJMJ24/SvJMJ23* (134 vs.169) (**Figures 7C, D**). These results indicate that while the coding sequences are conserved, the cis-regulatory landscapes of JmjC genes have undergone substantial remodeling, leading to species-specific regulatory patterns.

### Expression analysis of JmjC genes in different tissues at different developmental stages in foxtail millet and green foxtail

**Figure 8:**
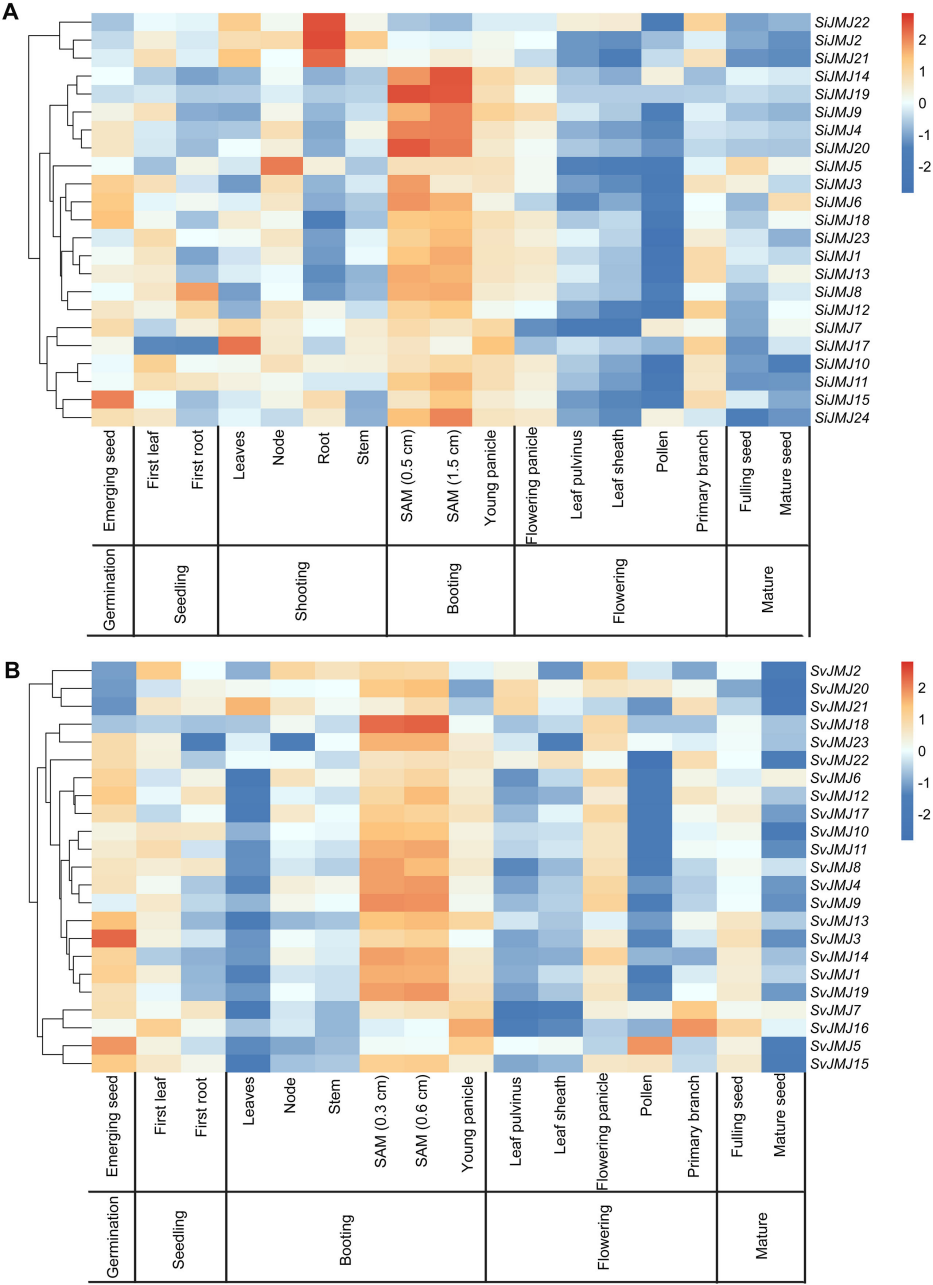
Spatiotemporal expression patterns of *SiJMJ* and *SvJMJ* genes across various tissues and developmental stages. **(A)** Expression profiles of *SiJMJ* genes in *Setaria italica*. **(B)** Expression profiles of *SvJMJ* genes in *Setaria viridis*. The heatmaps were constructed based on log2-transformed FPKM values from RNA-seq data. The color scale on the right represents row-scaled Z-scores, ranging from blue (low expression) to red (high expression). Genes were clustered hierarchically based on their expression patterns, as shown by the dendrogram on the left. The developmental stages at the bottom include germination, seedling, shooting, booting, flowering, and maturity. Key tissues such as leaves, roots, stems, shoot apical meristems (SAM), panicles, and seeds are indicated.

To explore the spatiotemporal expression patterns and potential biological functions of JmjC genes during plant development, we analyzed RNA-seq data from various tissues (including roots, stems, leaves, shoot apical meristems, panicles, and seeds) of *S.italica* (**Figure 8A**; **Supplementary Table 9**) and *S.viridis* (**Figure 8B**; **Supplementary Table 10**). Tissue-specific expression profiles in foxtail millet (*S.italica*) In *S.italica*, the *SiJMJ* genes exhibited distinct tissue-specific expression patterns and were clustered into three major groups based on hierarchical clustering (**Figure 8A**). A distinct subset of genes showed preferential expression in seed-related tissues. Notably, *SiJMJ15* exhibited exceptionally high expression levels specifically in emerging seeds (germination stage). A large cluster of genes, including *SiJMJ4*, *SiJMJ9*, *SiJMJ14*, *SiJMJ19*, and *SiJMJ20*, showed peak expression in the shoot apical meristem (SAM) (both 0.5 cm and 1.5 cm stages) and young panicles. Several genes displayed high expression in vegetative organs. For instance, *SiJMJ2*, *SiJMJ21* and *SiJMJ22* were predominantly expressed in roots during the shooting stage. Other genes like *SiJMJ17* showed specific upregulation in leaves.

Conservation of expression patterns in green foxtail (*S.viridis*) Analysis of the wild progenitor *S.viridis* revealed a largely conserved expression landscape (**Figure 8B**), reinforcing the functional stability of the JMJ family. Similar to foxtail millet, *SvJMJ* genes were also grouped into tissue-specific clusters. Genes such as *SvJMJ9*, *SvJMJ18*, *SvJMJ19* and *SvJMJ23* were highly enriched in SAM (0.3 cm and 0.6 cm) and young panicles. Additionally, *SvJMJ3* and *SvJMJ5* displayed high expression in emerging seeds. Overall, the comparative expression analysis demonstrates that *JmjC* genes in both *Setaria* species possess distinct and highly conserved spatiotemporal expression signatures. The dominant expression of specific subgroups in meristems (SAM) and reproductive tissues (panicles/seeds) strongly supports the hypothesis that these epigenetic modifiers are pivotal regulators of plant development and reproduction.

### Prediction of secondary and tertiary structures of JmjC proteins

To characterize the structural properties of the identified JMJ proteins, we analyzed their secondary structural elements and predicted their three-dimensional (3D) tertiary structures. The secondary structure analysis of the 24 *SiJMJ* proteins and 23 *SvJMJ* proteins revealed that the proteins are composed of alpha-helices, beta-sheets, random coils, and disordered regions (**Supplementary Figure 1**). In both *S.italica* and *S.viridis*, random coils and alpha-helices were identified as the predominant structural components across the gene family. For instance, in the stacked bar charts, the “Coil” (gray) and “Helix” (blue) segments consistently occupied the largest proportions of the total sequence length for most proteins, such as *SiJMJ2* and *SvJMJ23*. While extended strands (beta-sheets, orange) and disordered regions (yellow) were present, they generally constituted a smaller percentage of the overall structure compared to helices and coils. The structural composition patterns were highly similar between orthologous pairs (e.g., *SiJMJ1* vs. *SvJMJ1*), suggesting a conserved structural framework.

The 3D tertiary structures of the SiJMJ and SvJMJ proteins were predicted to further investigate their spatial folding (**Supplementary Figure 2**). The modeling results demonstrated that all identified proteins folded into distinct globular structures. Despite variations in protein length and loop regions, the core catalytic domains appeared structurally conserved. A comparative analysis between *S.italica* and *S.viridis* orthologs revealed a high degree of structural similarity. As shown in **Supplementary Figure 2**, the predicted 3D models of orthologous pairs, such as SiJMJ6 and SvJMJ6, or SiJMJ11 and SvJMJ11-exhibited nearly identical spatial conformations and folding patterns. This structural conservation, consistent with the secondary structure analysis, implies that the JmjC proteins in foxtail millet and green foxtail have maintained their functional structural architecture during evolution.

### Expression analysis of *SiJMJ* genes under different abiotic stresses

**Figure 9:**
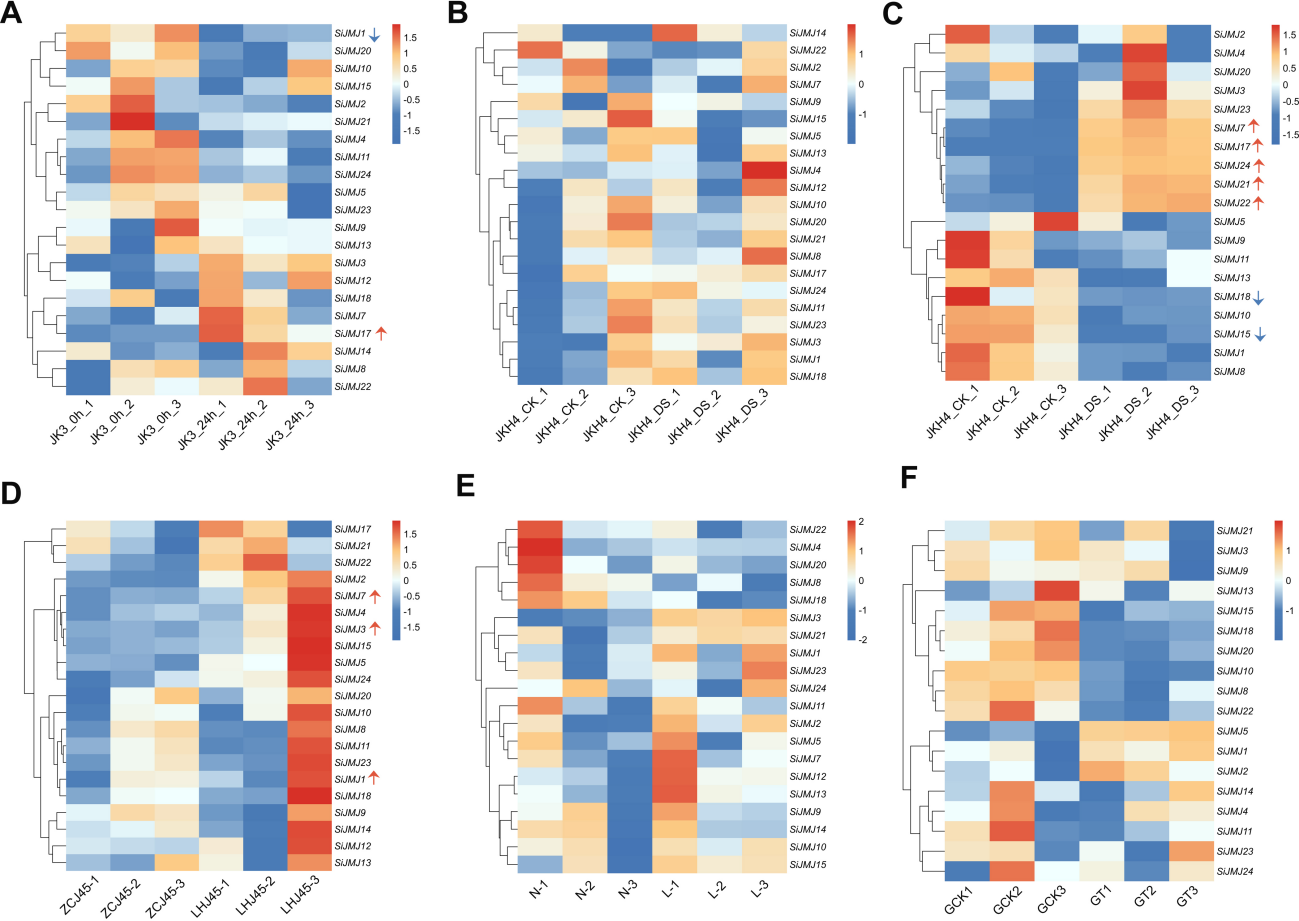
Expression profiles of *SiJMJ* genes under six different abiotic stresses. The heatmaps illustrate the expression patterns of *SiJMJ* genes based on RNA-seq data (log2-transformed FPKM values). **(A)** Expression profiles under saline-alkali stress. **(B)** Expression profiles under drought stress. **(C)** Expression profiles under low-temperature stress. **(D)** Expression profiles under waterlogging stress. **(E)** Expression profiles under low-nitrogen stress. **(F)** Expression profiles under herbicide stress. The color scale represents relative expression levels, ranging from blue (low) to red (high). Red upward arrows indicate upregulated genes, and blue downward arrows indicate downregulated genes in response to the respective stresses.

To investigate the transcriptional responses of the *SiJMJ* gene family to various environmental stimuli, we analyzed the expression profiles of all identified *SiJMJ* genes under saline-alkali, drought, cold, low nitrogen, and herbicide stresses using RNA-seq data (**Figure 9**). Under saline-alkali stress, the *SiJMJ* genes displayed distinct differential expression patterns (**Figure 9A**; **Supplementary Table 11**). As indicated by the red arrow, *SiJMJ17* was significantly upregulated after 24 hours of treatment compared to the control (0h), suggesting it plays a positive role in the response to salt stress. Conversely, *SiJMJ1* (marked with a blue arrow) showed a clear downregulation trend, indicating that their expression is repressed by saline-alkali conditions. In the drought stress treatment (JKH4_CK vs. JKH4_DS), the gene family exhibited a divergent response (**Figure 9B**). However, among the differentially expressed genes (DEGs) (**Supplementary Table 12**), we did not identify any significantly upregulated or downregulated genes. Under low temperature stress, compared with those in the control, seven DEGs (five upregulated and two downregulated) were differentially expressed after 24 h of low-temperature stress (**Figure 9C**; **Supplementary Table 13**). Specifically, *SiJMJ7*, *SiJMJ17*, *SiJMJ21*, *SiJMJ22*, and *SiJMJ24* were upregulated, whereas *SiJMJ15*and *SiJMJ18* were downregulated. These results suggest that these genes may be candidate genes associated with low-temperature stress response. Under waterlogging stress, a total of 21 *JMJ* genes were detected (**Figure 9D**; **Supplementary Table 14**). The results revealed that *SiJMJ1*, *SiJMJ3*, and *SiJMJ7* were significantly upregulated. Under low-nitrogen stress, a total of 20 expressed *JMJ* genes were detected (**Figure 9E**; **Supplementary Table 15**). Differential expression analysis was performed between the normal N and low N samples. Although several JMJ genes exhibited nonsignificant expression changes, no genes identified as significantly downregulated. Under atrazine stress, a total of 18 expressed *JMJ* genes were detected (**Figure 9F**; **Supplementary Table 16**). Compared with those of the control, a major cluster, including *SiJMJ1*, *SiJMJ2*, and *SiJMJ5*, showed high expression levels. Conversely, genes such as *SiJMJ8*, *SiJMJ10*, *SiJMJ15*, *SiJMJ18*, *SiJMJ20*, and *SiJMJ22* transitioned from moderate expression to low expression. In summary, the differential expression patterns observed under various stress conditions highlight the functional diversity of the *SiJMJ* family, identifying key candidate genes, such as *SiJMJ1*, *SiJMJ7*, and *SiJMJ17*, that may play critical roles in regulating abiotic stress responses in foxtail millet.

### Expression analysis of *SvJMJ* genes under heat stress

**Figure 10:**
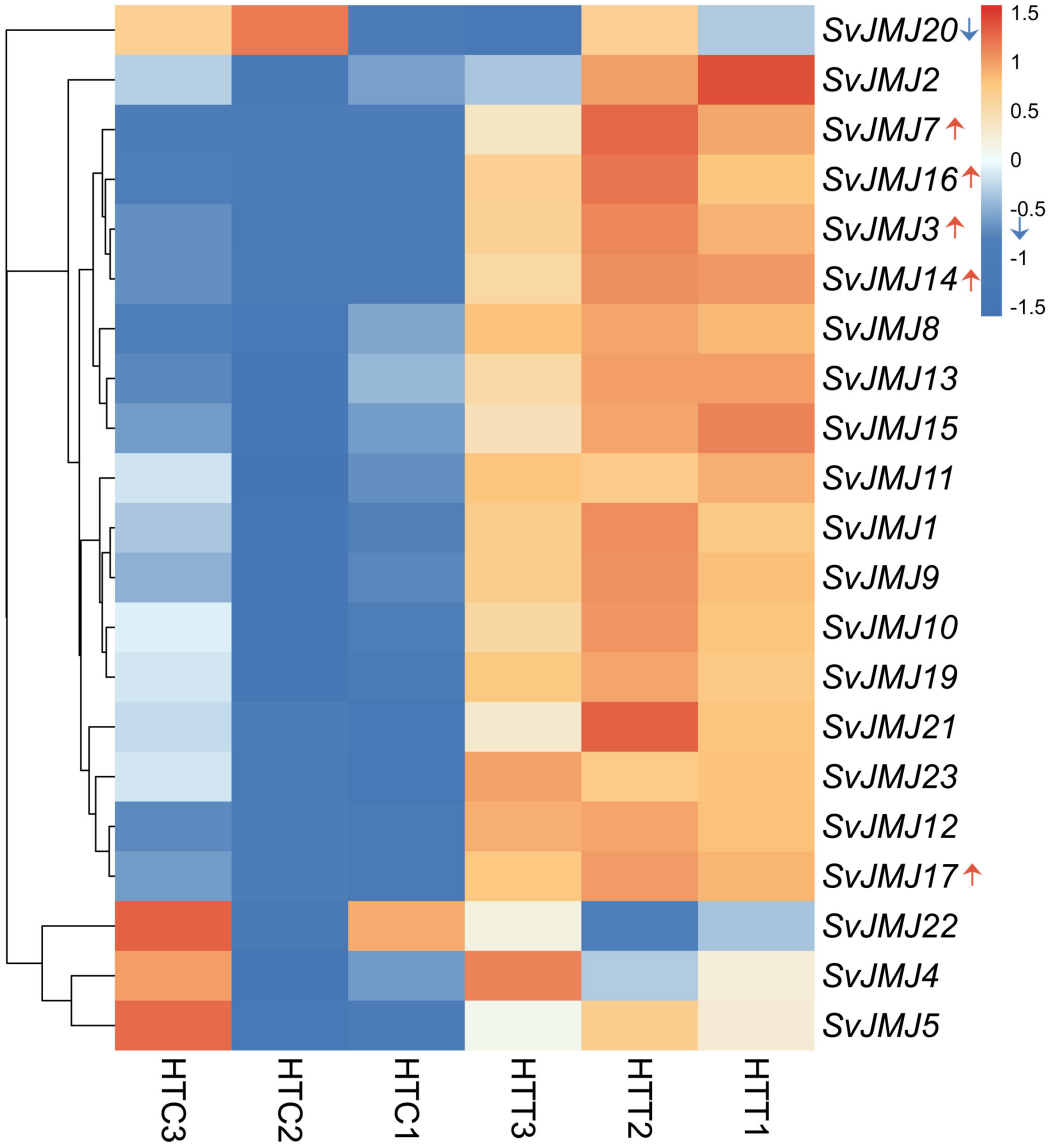
Expression profiles of *SvJMJ* genes under heat stress. The heatmap displays the expression patterns of *SvJMJ* genes in *Setaria viridis* leaves under control (HTC1–3) and heat stress (42 °C/32 °C, HTT1–3) conditions based on RNA-seq data (PRJNA896540). The color scale on the right represents the relative expression levels (row-scaled Z-score), ranging from blue (low) to red (high). Red upward arrows indicate genes significantly upregulated by heat stress (*SvJMJ7*, *SvJMJ16*, *SvJMJ3*, *SvJMJ14*, *SvJMJ17*), while the blue downward arrow indicates a downregulated gene (*SvJMJ20*).

The expression profiles of *SvJMJ* genes under heat stress were visualized using a hierarchically clustered heatmap (**Figure 10**; **Supplementary Table 17**). The analysis revealed distinct expression patterns between the control group (HTC) and the heat-stress group (HTT), suggesting extensive transcriptional reprogramming of the *SvJMJ* family in response to high temperatures.

As shown in **Figure 10**, the majority of *SvJMJ* genes exhibited differential expression. A specific cluster of genes, including *SvJMJ7*, *SvJMJ16*, *SvJMJ3*, and *SvJMJ14*, showed sharp upregulation under heat stress (indicated by red upward arrows), transitioning from low expression levels (blue) in the control samples to high expression levels (red) in the heat-treated samples. *SvJMJ17* was also induced by heat stress, although to a lesser extent compared to the highly upregulated cluster. In contrast, *SvJMJ20* was significantly downregulated (indicated by a blue downward arrow), suggesting a negative regulatory role or sensitivity to heat damage. Other genes, such as *SvJMJ2*, also displayed increased transcript abundance under heat conditions.

## Discussion

### Evolutionary conservation and lineage-specific adaptations of the JMJ family in Setaria

This study presents the first comprehensive genome-wide analysis of the JmjC domain-containing histone demethylase (JMJ) gene family in foxtail millet (*S.italica*) and its wild progenitor green foxtail (*S.viridis*). The identification of 24 and 23 JMJ genes in *S.italica* and *S.viridis*, respectively, reveals a gene family size comparable to that of other major angiosperms like rice (Wang et al., 2022) and Arabidopsis (Lu et al., 2008; Mistry et al., 2021). This consistency suggests that the core JMJ family, as a crucial component of the epigenetic machinery, has remained relatively stable throughout plant evolution without undergoing massive lineage-specific expansions. Phylogenetic classification into five conserved subfamilies (KDM5/JARID1, KDM4/JMDM3, KDM3/JMDM2, JMJD6, and JmjC-only) further underscores the ancient origin and functional conservation of this family, predating the monocot-dicot divergence (Han et al., 2016). The closer clustering of *Setaria* JMJ proteins with their rice orthologs, supported by extensive synteny (19 orthologous pairs), reinforces the shared evolutionary history within the Poaceae family and indicates that the fundamental epigenetic framework was established in a common cereal ancestor (Bennetzen et al., 2012). The near-perfect one-to-one orthology between *SiJMJ* and *SvJMJ* genes highlights remarkable genomic stability during the domestication of foxtail millet (Li and Brutnell, 2011). However, the identification of *Setaria*-specific sister gene pairs lacking direct rice orthologs, particularly within the KDM5/JARID1 and KDM3/JMDM2 subfamilies, points to subtle lineage-specific duplications and potential functional specializations after the divergence from the rice lineage.

### Structural stability contrasted with *cis*-regulatory divergence

A central finding of this work is the stark contrast between the high conservation of protein-coding sequences and the significant divergence in their cis-regulatory landscapes. Our structural analyses confirm that SiJMJ and SvJMJ proteins share highly conserved domain architectures, secondary structures, and predicted 3D folds, implying preserved enzymatic mechanisms for histone demethylation (Klose et al., 2006). This structural conservation is mirrored by the strong purifying selection (Ka/Ks<1) acting on the majority of orthologous and paralogous gene pairs. However, promoter analysis unveiled substantial rewiring. Wild *S.viridis* genes frequently possessed a higher density of stress-responsive *cis*-elements (e.g., MBS, LTR) and were predicted to be targeted by a greater number of transcription factors compared to their domesticated counterparts. A striking example is the *SvJMJ20/SiJMJ21* pair, where the wild gene’s promoter is targeted by 169 TFs versus only 70 for the cultivated gene. This reduction in regulatory complexity in foxtail millet aligns with the “domestication syndrome” hypothesis, where selection for uniform yield and stability in managed environments may have streamlined the dynamic, stress-responsive regulatory networks essential for survival in the wild (Meyer and Purugganan, 2013; Swinnen et al., 2016). Conversely, the signature of positive selection (Ka/Ks>1) detected in specific pairs like *SvJMJ18/SiJMJ19* and *SvJMJ22/SiJMJ23* suggests adaptive evolution at these loci, potentially contributing to agronomic traits selected during domestication.

### Spatiotemporal expression implicates roles in development and stress response

The expression profiles of *Setaria JMJ* genes exhibit strong and conserved tissue specificity between the two species. The preferential expression of genes like *SiJMJ4*, *SiJMJ9*, *SvJMJ9*, and *SvJMJ18* in the shoot apical meristem (SAM) and young panicles echoes the known functions of their homologs (e.g., *OsJMJ720 and OsJMJ706* in rice) in regulating reproductive development (Liu et al., 2014; Li et al., 2025). Similarly, the high specific expression of *SiJMJ15* and *SvJMJ15* in emerging seeds suggests a conserved role in seed biology, possibly in germination or dormancy regulation (Cho et al., 2012). These patterns underscore the integral role of JMJ-mediated demethylation in developmental phase transitions.

Furthermore, several *JMJ* genes were identified as responsive to abiotic stresses, aligning with foxtail millet’s renowned resilience. *SiJMJ17* emerged as a key multi-stress responder, showing significant upregulation under saline-alkali and cold stresses. This suggests it may act as a master epigenetic regulator, potentially removing repressive histone marks to activate broad-spectrum stress defense pathways, akin to ZmJMJ703 in maize (Wang et al., 2025). The expression of genes like *SiJMJ1* was highly context-dependent. While *SiJMJ1* was downregulated under salt stress, it was upregulated in response to cold, waterlogging, and herbicide treatments. This highlights the sophisticated nature of epigenetic modulation, which is tailored to specific environmental cues (Chang et al., 2020). The distinct induction of *SiJMJ1*, *SiJMJ2*, and *SiJMJ5* under herbicide (atrazine) stress points to a previously under-explored role for histone demethylation in regulating detoxification or herbicide response pathways in crops (Gaines et al., 2020). Mechanistically, we hypothesize that these JMJ proteins may function by removing repressive histone marks (such as H3K27me3 or H3K9me2) from the promoters of key xenobiotic detoxification genes, particularly Cytochrome P450 monooxygenases (P450s) (Rong Tan et al., 2015) and Glutathione S-transferases (GSTs) (Nakka et al., 2017). This epigenetic depression would facilitate the rapid transcriptional activation of detoxification machinery, thereby enhancing the plant’s tolerance to chemical stress (Markus et al., 2018).

## Conclusion

In summary, this study provides a foundational resource for the gene family in the *Setaria* genus. We demonstrate that while the structural core of JMJ proteins is evolutionarily constrained, their transcriptional regulatory networks have undergone significant remodeling, likely influenced by domestication. The identification of stress-responsive candidates, particularly *SiJMJ17*, offers promising targets for epigenetic engineering to enhance abiotic stress tolerance in cereals. Future work should focus on functional validation using CRISPR/Cas9 or RNAi approaches in *Setaria* to definitively establish the roles of these candidate genes. Additionally, profiling histone methylation dynamics at target genes under stress in wild-type and mutant plants will be crucial to elucidate the precise epigenetic mechanisms governed by these demethylases.

## Data Availability

The original contributions presented in the study are included in the article/**Supplementary Material**. Further inquiries can be directed to the corresponding authors.

## References

[B1] Bennetzen J. L. Schmutz J. Wang H. Percifield R. Hawkins J. Pontaroli A. C. . (2012). Reference genome sequence of the model plant Setaria. Nat. Biotechnol. 30, 555–561. doi: 10.1038/nbt.2196. PMID: 22580951

[B2] Blum M. Andreeva A. Florentino L. C. Chuguransky S. R. Grego T. Hobbs E. . (2025). InterPro: the protein sequence classification resource in 2025. Nucleic Acids Res. 53, D444–DD56. doi: 10.1093/nar/gkae1082. PMID: 39565202 PMC11701551

[B3] Brutnell T. P. Wang L. Swartwood K. Goldschmidt A. Jackson D. Zhu X. G. . (2010). Setaria viridis: a model for C4 photosynthesis. Plant Cell 22, 2537–2544. doi: 10.1007/978-3-319-45105-3_17. PMID: 20693355 PMC2947182

[B4] Chai J. Gu X. Song P. Zhao X. Gao Y. Wang H. . (2024). Histone demethylase JMJ713 interaction with JMJ708 modulating H3K36me2, enhances rice heat tolerance through promoting hydrogen peroxide scavenging. Plant Physiol. Biochem. 217, 109284. doi: 10.1016/j.plaphy.2024.109284. PMID: 39536507

[B5] Chang X. Zhang S. Cao C. Zhou J. Wang X. Zhang D. . (2024). Transcriptome analysis and characteristics of drought resistance related genes in four varieties of foxtail millet [Setaria italica. Heliyon 10, e38083. doi: 10.1016/j.heliyon.2024.e38083. PMID: 39364255 PMC11447331

[B6] Chang Y. N. Zhu C. Jiang J. Zhang H. Zhu J. K. Duan C. G. (2020). Epigenetic regulation in plant abiotic stress responses. J. Integr. Plant Biol. 62, 563–580. doi: 10.1111/jipb.12901. PMID: 31872527

[B7] Chao J. Li Z. Sun Y. Aluko O. O. Wu X. Wang Q. . (2021). MG2C: a user-friendly online tool for drawing genetic maps. Mol. Hortic. 1, 16. doi: 10.1186/s43897-021-00020-x. PMID: 37789491 PMC10514940

[B8] Chen C. Wu Y. Li J. Wang X. Zeng Z. Xu J. . (2023). TBtools-II: A "one for all, all for one" bioinformatics platform for biological big-data mining. Mol. Plant 16, 1733–1742. doi: 10.1016/j.molp.2023.09.010. PMID: 37740491

[B9] Cho J. N. Ryu J. Y. Jeong Y. M. Park J. Song J. J. Amasino R. M. . (2012). Control of seed germination by light-induced histone arginine demethylation activity. Dev. Cell 22, 736–748. doi: 10.1016/j.devcel.2012.01.024. PMID: 22483719

[B10] Eddy S. R. (2011). Accelerated profile HMM searches. PloS Comput. Biol. 7, e1002195. doi: 10.1371/journal.pcbi.1002195. PMID: 22039361 PMC3197634

[B11] Gaines T. A. Duke S. O. Morran S. Rigon C. A. G. Tranel P. J. Kupper A. . (2020). Mechanisms of evolved herbicide resistance. J. Biol. Chem. 295, 10307–10330. doi: 10.1074/jbc.rev120.013572. PMID: 32430396 PMC7383398

[B12] Goodstein D. M. Shu S. Howson R. Neupane R. Hayes R. D. Fazo J. . (2012). Phytozome: a comparative platform for green plant genomics. Nucleic Acids Res. 40, D1178–D1186. doi: 10.1093/nar/gkr944. PMID: 22110026 PMC3245001

[B13] Han Y. Li X. Cheng L. Liu Y. Wang H. Ke D. . (2016). Genome-wide analysis of soybean JmjC domain-containing proteins suggests evolutionary conservation following whole-genome duplication. Front. Plant Sci. 7. doi: 10.3389/fpls.2016.01800. PMID: 27994610 PMC5136575

[B14] He Q. Wang C. He Q. Zhang J. Liang H. Lu Z. . (2024). A complete reference genome assembly for foxtail millet and Setaria-db, a comprehensive database for Setaria. Mol. Plant 17, 219–222. doi: 10.1016/j.molp.2023.12.017. PMID: 38155573

[B15] Hou Y. Wang L. Wang L. Liu L. Li L. Sun L. . (2015). JMJ704 positively regulates rice defense response against Xanthomonas oryzae pv. oryzae infection via reducing H3K4me2/3 associated with negative disease resistance regulators. BMC Plant Biol. 15, 286. doi: 10.1186/s12870-015-0674-3. PMID: 26646110 PMC4673860

[B16] Jia G. Huang X. Zhi H. Zhao Y. Zhao Q. Li W. . (2013). A haplotype map of genomic variations and genome-wide association studies of agronomic traits in foxtail millet (Setaria italica). Nat. Genet. 45, 957–961. doi: 10.1038/ng.2673. PMID: 23793027

[B17] Jones M. A. Morohashi K. Grotewold E. Harmer S. L. (2019). Arabidopsis JMJD5/JMJ30 acts independently of LUX ARRHYTHMO within the plant circadian clock to enable temperature compensation. Front. Plant Sci. 10. doi: 10.3389/fpls.2019.00057. PMID: 30774641 PMC6367231

[B18] Khator K. Parihar S. Jasik J. Shekhawat G. S. (2024). Nitric oxide in plants: an insight on redox activity and responses toward abiotic stress signaling. Plant Signal. Behav. 19, 2298053. doi: 10.1080/15592324.2023.2298053. PMID: 38190763 PMC10793691

[B19] Kim J. M. Sasaki T. Ueda M. Sako K. Seki M. (2015). Chromatin changes in response to drought, salinity, heat, and cold stresses in plants. Front. Plant Sci. 6. doi: 10.3389/fpls.2015.00114. PMID: 25784920 PMC4345800

[B20] Klose R. J. Kallin E. M. Zhang Y. (2006). JmjC-domain-containing proteins and histone demethylation. Nat. Rev. Genet. 7, 715–727. doi: 10.1038/nrg1945. PMID: 16983801

[B21] Kumar S. Stecher G. Suleski M. Sanderford M. Sharma S. Tamura K. (2024). MEGA12: Molecular evolutionary genetic analysis version 12 for adaptive and green computing. Mol. Biol. Evol. 41, msae263. doi: 10.1093/molbev/msae263. PMID: 39708372 PMC11683415

[B22] Le H. Simmons C. H. Zhong X. (2025). Functions and mechanisms of histone modifications in plants. Annu. Rev. Plant Biol. 76, 551–578. doi: 10.1146/annurev-arplant-083123-070919. PMID: 39952674 PMC13276730

[B23] Lescot M. Dehais P. Thijs G. Marchal K. Moreau Y. Van de Peer Y. . (2002). PlantCARE, a database of plant cis-acting regulatory elements and a portal to tools for in silico analysis of promoter sequences. Nucleic Acids Res. 30, 325–327. doi: 10.1093/nar/30.1.325. PMID: 11752327 PMC99092

[B24] Letunic I. Khedkar S. Bork P. (2021). SMART: recent updates, new developments and status in 2020. Nucleic Acids Res. 49, D458–DD60. doi: 10.1093/nar/gkaa937. PMID: 33104802 PMC7778883

[B25] Li P. Brutnell T. P. (2011). Setaria viridis and Setaria italica, model genetic systems for the Panicoid grasses. J. Exp. Bot. 62, 3031–3037. doi: 10.1093/jxb/err096. PMID: 21459768

[B26] Li X. Zhu J. Tian X. Liu Y. Wei J. Hong Z. . (2025). JMJ720 regulates flowering time in rice via H3K9 demethylation of Hd1. Plant Biotechnol. J. 23, 3824–3837. doi: 10.1111/pbi.70206. PMID: 40517394 PMC12392927

[B27] Liu H. Guo S. Xu Y. Li C. Zhang Z. Zhang D. . (2014). OsmiR396d-regulated OsGRFs function in floral organogenesis in rice through binding to their targets OsJMJ706 and OsCR4. Plant Physiol. 165, 160–174. doi: 10.1104/pp.114.235564. PMID: 24596329 PMC4012577

[B28] Love M. I. Huber W. Anders S. (2014). Moderated estimation of fold change and dispersion for RNA-seq data with DESeq2. Genome Biol. 15, 550. doi: 10.1186/s13059-014-0550-8. PMID: 25516281 PMC4302049

[B29] Lu F. Li G. Cui X. Liu C. Wang X. J. Cao X. (2008). Comparative analysis of JmjC domain-containing proteins reveals the potential histone demethylases in Arabidopsis and rice. J. Integr. Plant Biol. 50, 886–896. doi: 10.1111/j.1744-7909.2008.00692.x. PMID: 18713399

[B30] Lu S. X. Knowles S. M. Webb C. J. Celaya R. B. Cha C. Siu J. P. . (2011). The Jumonji C domain-containing protein JMJ30 regulates period length in the Arabidopsis circadian clock. Plant Physiol. 155, 906–915. doi: 10.1104/pp.110.167015. PMID: 21139085 PMC3032475

[B31] Lu S. Wang J. Chitsaz F. Derbyshire M. K. Geer R. C. Gonzales N. R. . (2020). CDD/SPARCLE: the conserved domain database in 2020. Nucleic Acids Res. 48, D265–D2D8. doi: 10.1093/nar/gkz991. PMID: 31777944 PMC6943070

[B32] Markus C. Pecinka A. Karan R. Barney J. N. Merotto A. (2018). Epigenetic regulation - contribution to herbicide resistance in weeds? Pest Manag Sci. 74, 275–281. doi: 10.1002/ps.4727. PMID: 28888062

[B33] Meyer R. S. Purugganan M. D. (2013). Evolution of crop species: genetics of domestication and diversification. Nat. Rev. Genet. 14, 840–852. doi: 10.1038/nrg3605. PMID: 24240513

[B34] Mistry J. Chuguransky S. Williams L. Qureshi M. Salazar G. A. Sonnhammer E. L. L. . (2021). Pfam: The protein families database in 2021. Nucleic Acids Res. 49, D412–D4D9. doi: 10.1093/nar/gkaa913. PMID: 33125078 PMC7779014

[B35] Muthamilarasan M. Prasad M. (2021). Small millets for enduring food security amidst pandemics. Trends Plant Sci. 26, 33–40. doi: 10.1016/j.tplants.2020.08.008. PMID: 32900620 PMC7474701

[B36] Nakka S. Godar A. S. Thompson C. R. Peterson D. E. Jugulam M. (2017). Rapid detoxification via glutathione S-transferase (GST) conjugation confers a high level of atrazine resistance in Palmer amaranth (Amaranthus palmeri). Pest Manag Sci. 73, 2236–2243. doi: 10.1002/ps.4615. PMID: 28500680

[B37] Nie Y. Guo L. Cui F. Shen Y. Ye X. Deng D. . (2022). Innovations and stepwise evolution of CBFs/DREB1s and their regulatory networks in angiosperms. J. Integr. Plant Biol. 64, 2111–2125. doi: 10.1111/jipb.13357. PMID: 36070250

[B38] Qian Y. Chen C. Jiang L. Zhang J. Ren Q. (2019). Genome-wide identification, classification and expression analysis of the JmjC domain-containing histone demethylase gene family in maize. BMC Genomics 20, 256. doi: 10.1186/s12864-019-5633-1. PMID: 30935385 PMC6444447

[B39] Rong Tan L. Chen Lu Y. Jing Zhang J. Luo F. Yang H. (2015). A collection of cytochrome P450 monooxygenase genes involved in modification and detoxification of herbicide atrazine in rice (Oryza sativa) plants. Ecotoxicol. Environ. Saf. 119, 25–34. doi: 10.1016/j.ecoenv.2015.04.035. PMID: 25968601

[B40] Subramanian B. Gao S. Lercher M. J. Hu S. Chen W. H. (2019). Evolview v3: a webserver for visualization, annotation, and management of phylogenetic trees. Nucleic Acids Res. 47, W270–W2W5. doi: 10.1093/nar/gkz357. PMID: 31114888 PMC6602473

[B41] Sun L. Liu L. Wang Y. Feng Y. Yang W. Wang D. . (2022). Integration of metabolomics and transcriptomics for investigating the tolerance of foxtail millet (Setaria italica) to atrazine stress. Front. Plant Sci. 13. doi: 10.3389/fpls.2022.890550. PMID: 35755691 PMC9226717

[B42] Swinnen G. Goossens A. Pauwels L. (2016). Lessons from domestication: targeting cis-regulatory elements for crop improvement. Trends Plant Sci. 21, 506–515. doi: 10.1016/j.tplants.2019.09.004. PMID: 26876195

[B43] Tian F. Yang D. C. Meng Y. Q. Jin J. Gao G. (2020). PlantRegMap: charting functional regulatory maps in plants. Nucleic Acids Res. 48, D1104–D1D13. doi: 10.1093/nar/gkz1020. PMID: 31701126 PMC7145545

[B44] Wang S. Jiang L. Zhai T. Qu K. Liu X. Di Z. . (2025). The JmjC domain-containing histone demethylase ZmJMJ703 orchestrates salt stress adaptation in maize. J. Plant Physiol. 317, 154677. doi: 10.1016/j.jplph.2025.154677. PMID: 41418723

[B45] Wang X. Pan C. Long J. Bai S. Yao M. Chen J. . (2022). Genome-wide identification of the jumonji C domain- containing histone demethylase gene family in wheat and their expression analysis under drought stress. Front. Plant Sci. 13. doi: 10.3389/fpls.2022.987257. PMID: 36092409 PMC9453444

[B46] Yang Y. Li Y. Guo Z. Zhao Y. Zhou X. Han Y. . (2025). Identification of DREB gene family in foxtail millet (Setaria italica) and analysis of its expression pattern in response to abiotic stress. Front. Plant Sci. 16. doi: 10.3389/fpls.2025.1552120. PMID: 40357163 PMC12066435

[B47] Zhang H. Lang Z. Zhu J. K. (2018). Dynamics and function of DNA methylation in plants. Nat. Rev. Mol. Cell Biol. 19, 489–506. doi: 10.1038/s41580-018-0016-z. PMID: 29784956

[B48] Zhang P. Sharwood R. E. Carroll A. Estavillo G. M. von Caemmerer S. Furbank R. T. (2025). Systems analysis of long-term heat stress responses in the C4 grass Setaria viridis. Plant Cell 37. doi: 10.1093/plcell/koaf005. PMID: 39778116 PMC11964294

[B49] Zhu J. K. (2016). Abiotic stress signaling and responses in plants. Cell. 167, 313–324. doi: 10.1016/j.cell.2016.08.029. PMID: 27716505 PMC5104190

