# Supplementary material for: Genome-wide comparative analysis of the JmjC gene family in Setaria italica and Setaria viridis reveals transcriptional divergence and stress-responsive candidates
Source: Front Plant Sci. 2026 Mar 27;17:1793570. doi: 10.3389/fpls.2026.1793570 (PMC13066240; doi:10.3389/fpls.2026.1793570)
Supplement: Supplementary file 1 [file DataSheet1.pdf]

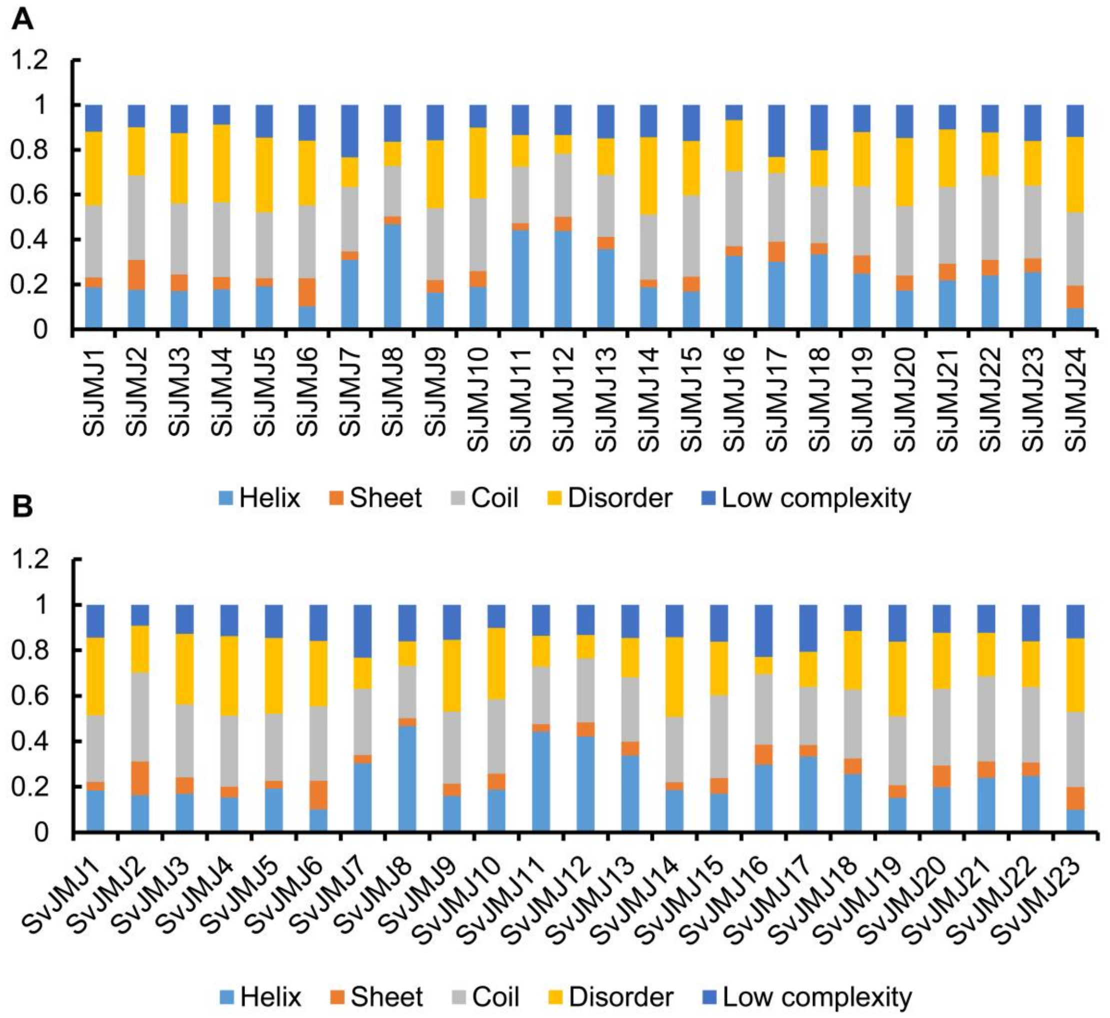

Supplementary Figure 1. Predicted secondary structure analysis of SiJMJ and SvJMJ proteins. The stacked bar charts illustrate the proportional composition of secondary structural elements in (A) *Setaria italica* (SiJMJs) and (B) *Setaria viridis* (SvJMJs). The distinct colored segments within each bar represent specific structural components: alpha-helix (light blue), extended strand/sheet (orange), random coil (gray), disordered region (yellow), and low-complexity region (dark blue). The y-axis indicates the ratio of each structural component relative to the total protein sequence length.

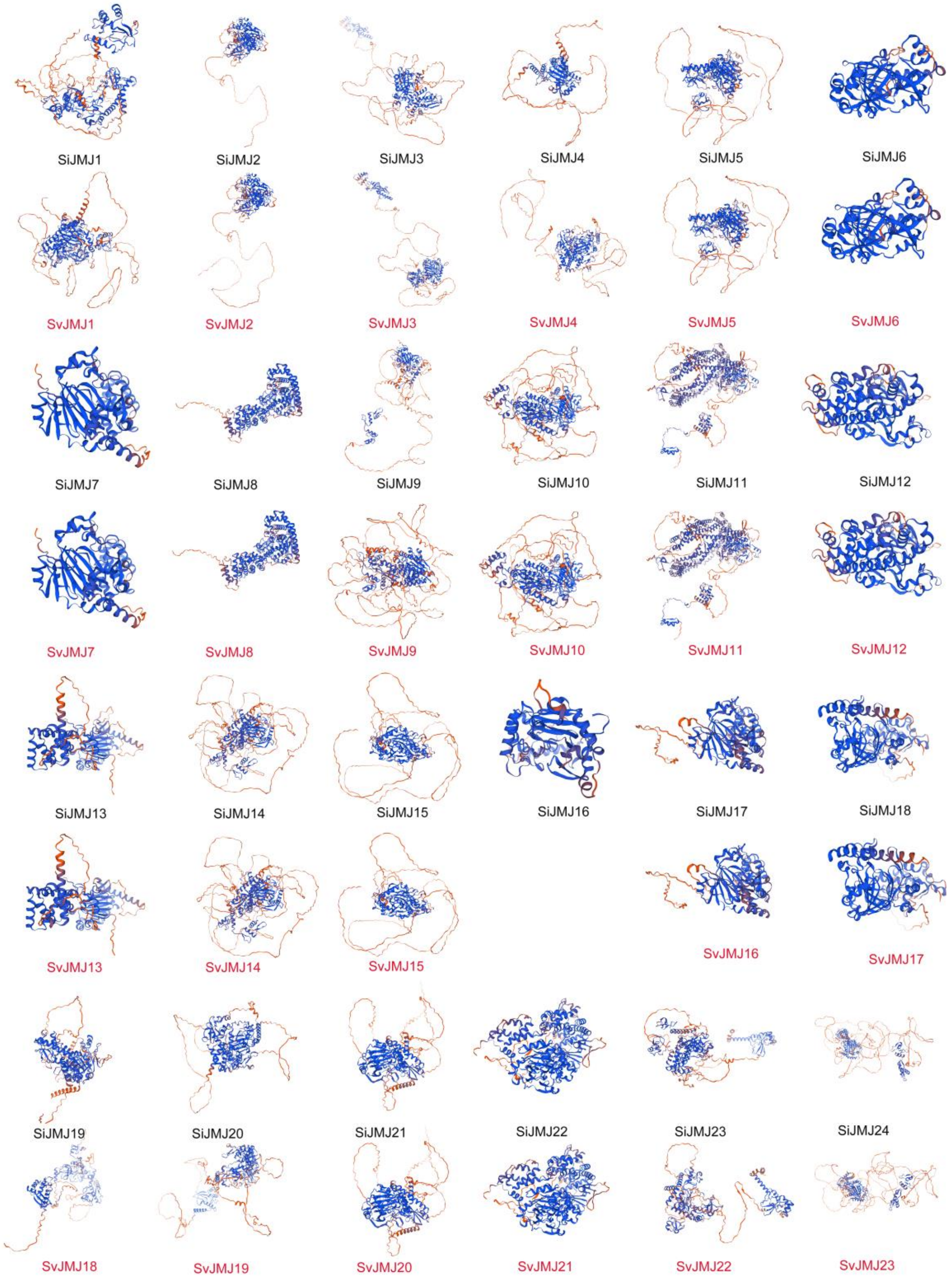

Supplementary Figure 2. Predicted tertiary structures of SiJMJ and SvJMJ proteins. The three-dimensional (3D) structural models of JmjC proteins were constructed using the SWISS-MODEL homology modeling server. The figure displays the predicted tertiary structures for JmjC family members in *Setaria italica* (SiJMJs, labeled in black) and their orthologs in *Setaria viridis* (SvJMJs, labeled in red). The protein structures are visualized in ribbon representation, highlighting the conserved folding patterns (shown in blue) and structural variations between the domesticated crop and its wild progenitor.
